# Computational prioritization of multi-target inhibitors: explainable QSAR and docking-based discovery of dual AChE/BACE1 chemotypes

**DOI:** 10.1007/s10822-025-00757-3

**Published:** 2026-01-28

**Authors:** İsa Bozkır, Merve Seda İbişoğlu, İlknur Kayıkçıoğlu Bozkır, Halil İbrahim Güler

**Affiliations:** 1 Department of Medical Services and Techniques, Gumushane Health Services Vocational School, Gumushane University, Gümüşhane, Turkey; 2 Department of Molecular Biology and Genetics, Graduate School of Natural and Applied Science, Karadeniz Technical University, 61080 Trabzon, Turkey; 3 Department of Molecular Biology and Genetics, Faculty of Science, Karadeniz Technical University, 61080 Trabzon, Turkey; 4 Department of Software Engineering, Faculty of Engineering and Natural Sciences, Gumushane University, Gümüşhane, Turkey

**Keywords:** Alzheimer’s disease, AChE, BACE1, Dual inhibitors, Explainable QSAR, Docking/ADMET

## Abstract

The discovery of dual acetylcholinesterase (AChE) and β-secretase (BACE1) inhibitors remains a promising strategy against multifactorial Alzheimer’s disease. Here, rigorously curated ChEMBL-derived data were used to develop explainable QSAR (Quantitative structure–activity relationship) models for dual-inhibition prioritization. Molecules were standardized, near-duplicates were removed using a Tanimoto similarity threshold (≥ 0.80), and physicochemical outliers were filtered prior to modeling. Multiple classifiers (including Light Gradient-Boosting Machine, eXtreme Gradient Boosting, Random Forest, Support Vector Machine, k-Nearest Neighbors and Gradient Boosting Decision Trees) and fingerprints (e.g., RDKit fingerprints, Extended Connectivity Fingerprint) were benchmarked under scaffold-based nested cross-validation to prevent data leakage. Class imbalance was handled with SMOTETomek applied strictly within training folds. Model selection relied on F-Score, Area Under the Precision–Recall Curve, Matthews Correlation Coefficient (MCC), and Recall, and performance was accompanied by bootstrap confidence intervals, calibration curves, and Y-randomization controls. In classification, the top model (GBDT + ECFP6) achieved strong generalization (Recall ≈ 1.00, PR-AUC ≈ 0.84, MCC ≈ 0.81, F1 Score ≈ 0.84). Shapley Additive Explanations (SHAP) analysis highlighted aromatic and hydrogen-bonding substructures as key positive contributors. Prospective candidates (e.g., CHEMBL5082250, CHEMBL1651126, CHEMBL1651127) were evaluated by active-site-focused docking against AChE (PDB: 4EY7) and BACE1 (PDB: 2G94) with essential waters retained; docking scores (ΔG, kcal·mol⁻^1^) were used for relative ranking of the ligands. SwissADME/pkCSM profiling suggested CNS-relevant properties (e.g., MPO, logBB, P-gp liability) and acceptable oral drug-likeness. Collectively, the workflow provides a reproducible and transparent pipeline for prioritizing dual AChE/BACE1 chemotypes and nominates testable scaffolds for experimental validation.

**Supplementary Information:**

The online version contains supplementary material available at 10.1007/s10822-025-00757-3.

## Introduction

Alzheimer’s disease (AD) is a progressive neurodegenerative disorder characterized by cognitive decline, memory loss, and synaptic dysfunction, ultimately leading to severe disability and death. Despite decades of research, current therapeutic interventions remain largely symptomatic, offering only temporary relief without altering disease progression. Among the multiple pathological mechanisms implicated in AD, cholinergic neurotransmission deficits and amyloid-beta (Aβ) peptide accumulation are two of the most prominent hallmarks. Acetylcholinesterase (AChE; EC 3.1.1.7) catalyzes the hydrolysis of acetylcholine in the synaptic cleft, thereby terminating cholinergic signaling. In AD, excessive AChE activity aggravates cholinergic deficits, contributing to memory impairment. In parallel, β-site amyloid precursor protein cleaving enzyme 1 (BACE1; EC 3.4.23.46) mediates the rate-limiting step of Aβ generation, leading to extracellular amyloid plaque formation—a pathological signature strongly associated with disease onset and progression [22,43, 48].

Given the multifactorial nature of AD, multi-target directed ligands (MTDLs) capable of modulating multiple pathological pathways simultaneously have emerged as a promising therapeutic strategy. In particular, dual inhibition of AChE and BACE1 holds the potential to address both the cholinergic deficit and amyloidogenic cascade. Unlike conventional single-target drug discovery, MTDL-based design may enhance therapeutic efficacy, reduce polypharmacy, and minimize adverse effects [20, 38]. Although several hybrid molecules and natural product derivatives have shown dual inhibitory activity, identifying potent, selective, and pharmacokinetically favorable candidates remains a formidable challenge [12, 41].

**Fig. 1:**
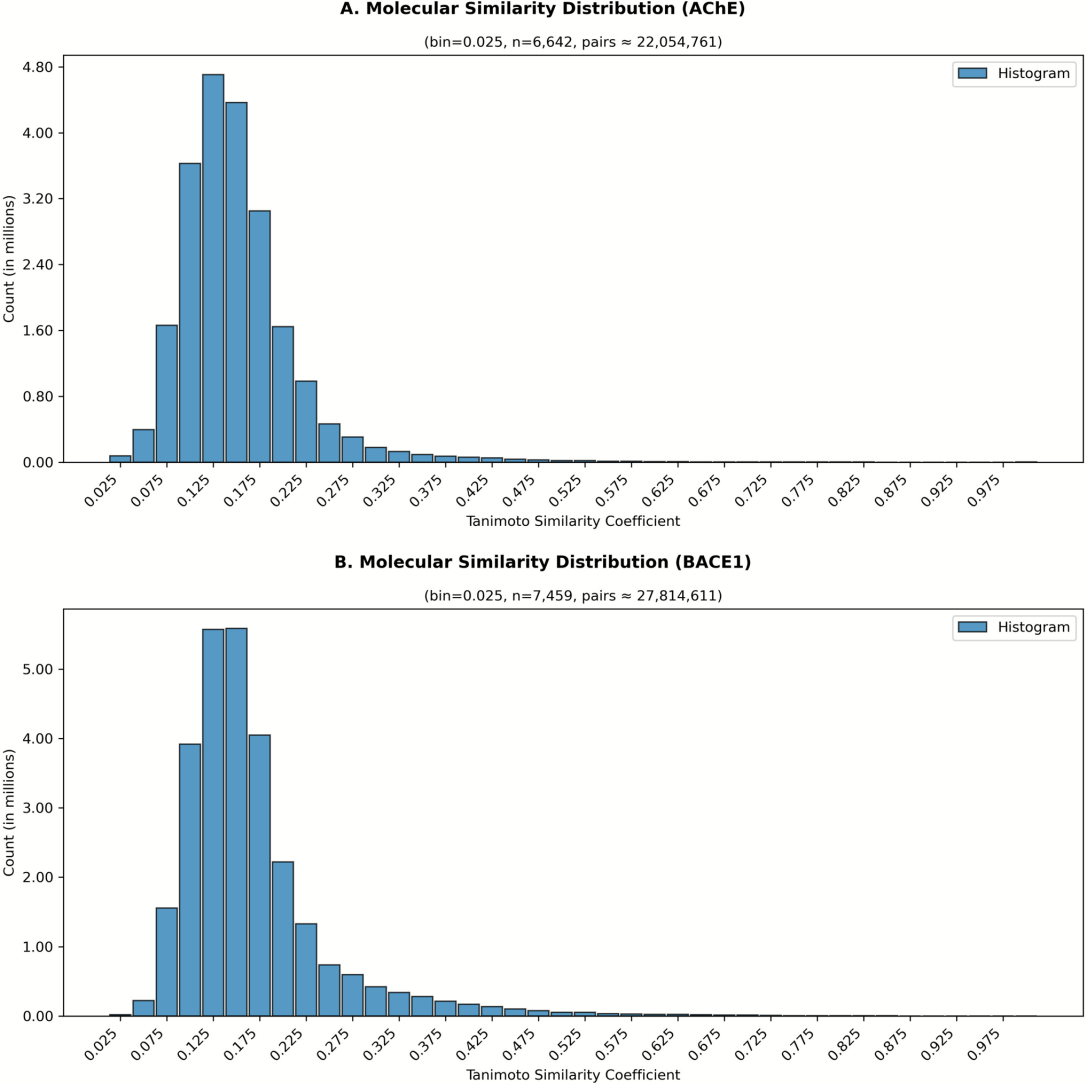
Structural similarity distributions of AChE and BACE1 datasets. Curated bioactivity datasets for acetylcholinesterase (AChE) (**A**) and β-site amyloid precursor protein cleaving enzyme 1 (BACE1) (**B**) were analyzed using ECFP6 molecular fingerprints

**Fig. 2:**
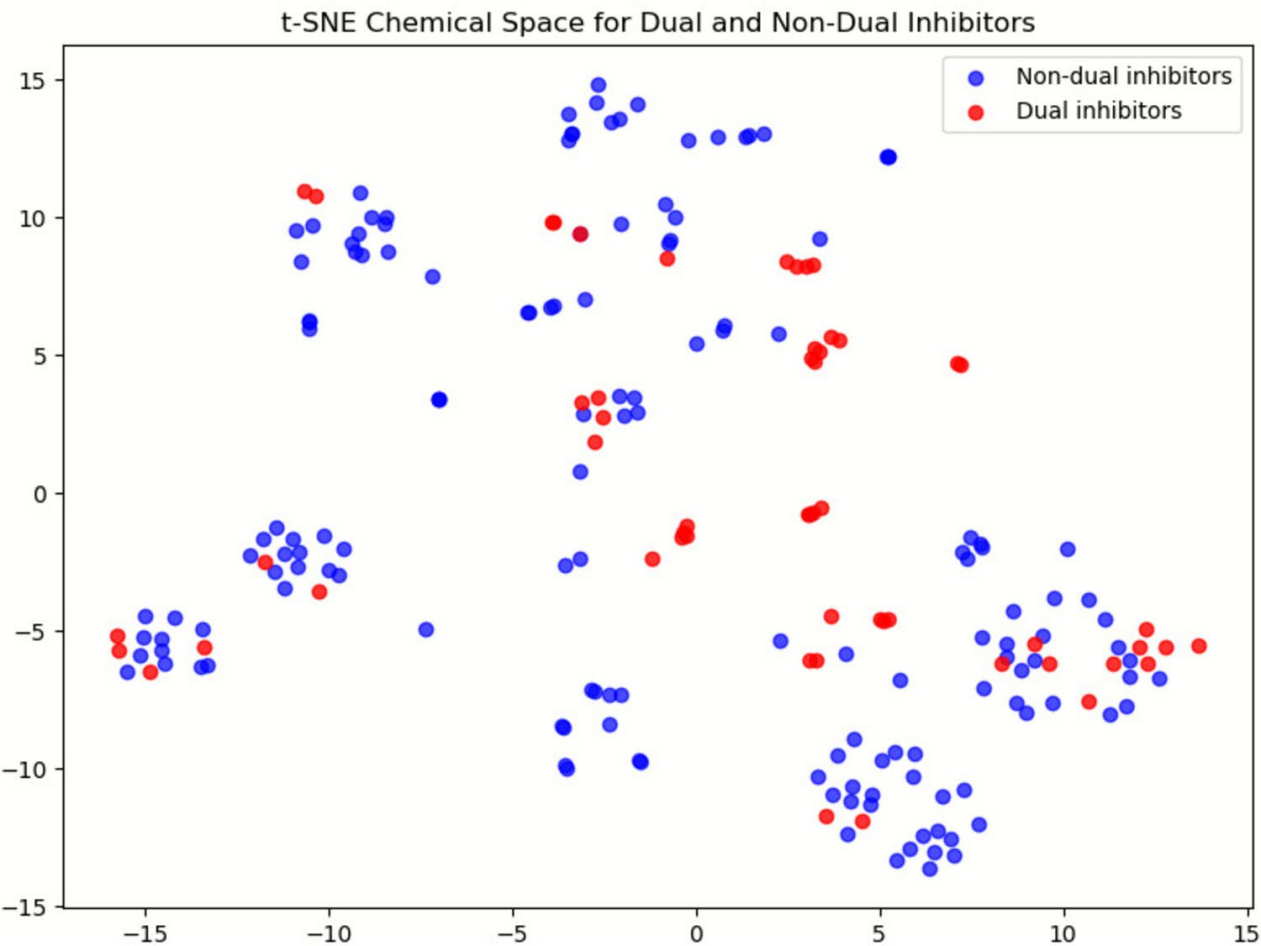
t-SNE projection of the ECFP6 chemical space for 204 compounds. Red points indicate dual inhibitors (n = 55) and blue points indicate non-dual inhibitors (n = 149). Although the average inter-class Tanimoto similarity is low (mean = 0.12; median = 0.09), the projection reveals partial overlap between the classes, reflecting the structural heterogeneity of the dataset and the inherent difficulty of the classification task

Recent advances in computational drug discovery—particularly machine learning (ML)-driven quantitative structure–activity relationship (QSAR) modeling—have enabled rapid, large-scale screening and optimization of bioactive molecules. By integrating diverse molecular descriptors, advanced feature selection algorithms, and high-performance ML models, QSAR approaches can predict biological activities of untested compounds with high accuracy, thus accelerating the discovery process while reducing experimental costs [6, 10]. Moreover, explainable artificial intelligence (XAI) techniques such as SHapley Additive exPlanations (SHAP) offer interpretable insights into the structural determinants of activity, facilitating rational multi-target drug design [32]. Recently, Dhamodharan and Mohan [14] developed ligand-based machine learning (QSAR/ANN/SVM) models for dual inhibition of AChE and BACE1, using a set of structural, electro-topological and spatial descriptors, and achieved good predictive performance (R^2^ ~ 0.82–0.87, q^2^ ~ 0.78–0.86) for both targets. While their work demonstrates the feasibility of ML-driven dual-inhibitor design, it remains limited to ligand-based descriptors and lacks explicit evaluation of 3D binding modes, ADMET properties, and interpretability analysis via SHAP or other XAI methods. In contrast, the present study addresses these gaps by combining docking-based binding-mode evaluation, ADMET and toxicity prediction, and explainable AI approaches to assess ligand–target interactions and drug-like suitability [14] (Figs. 1, 2).

**Fig. 3:**
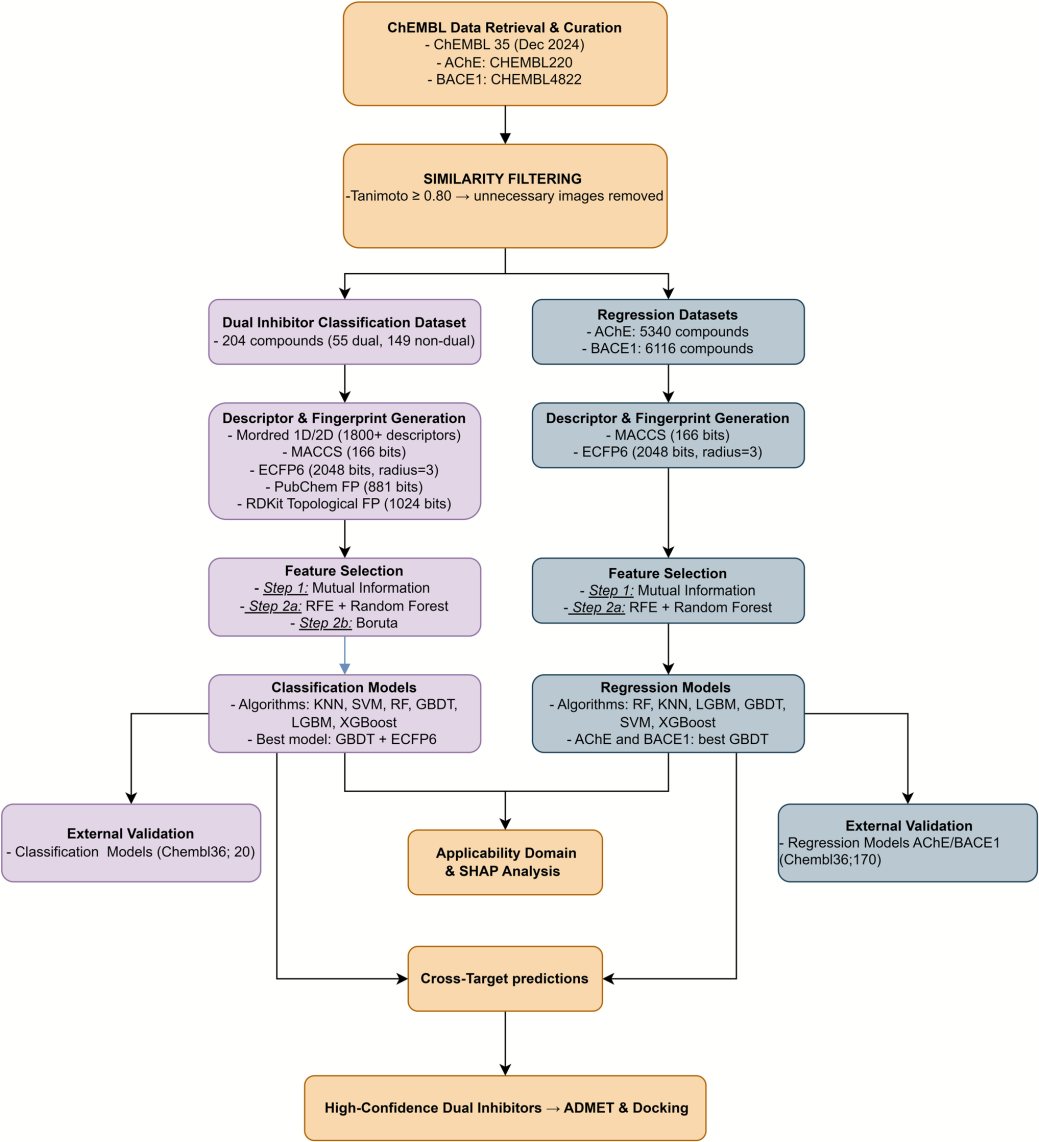
Overall workflow used to identify dual AChE/BACE1 inhibitors

In this study, we developed an integrative cheminformatics and molecular modeling framework to identify potential dual AChE/BACE1 inhibitors. Following systematic dataset curation from ChEMBL, structural filtering, and descriptor generation, multiple ML classification and regression models were constructed and evaluated. The best-performing models were interpreted using SHAP to pinpoint structural motifs associated with dual inhibition, and high-confidence predictions were subsequently validated via molecular docking against high-resolution AChE (PDB ID: 4EY7) and BACE1 (PDB ID: 2G94) structures. This comprehensive strategy not only highlights promising dual inhibitors but also delineates their key activity-driving features, providing a blueprint for the rational design of next-generation therapeutics in Alzheimer’s disease. The overall workflow of the study is summarized in Fig. 3.

## Material and methods

### Dataset acquisition and preprocessing

Bioactivity datasets for *Homo sapiens* acetylcholinesterase (AChE; ChEMBL target ID: CHEMBL220) and β-site amyloid precursor protein cleaving enzyme 1 (BACE1; ChEMBL target ID: CHEMBL4822) were retrieved from the ChEMBL database [17]. Bioactivity records were obtained using the chembl-webresource-client Python interface, accessing the ChEMBL 35 database (December 2024 release), which represented the most recent version available at the time the study was conducted. Only experimental IC_50_ measurements were retained. To ensure data quality, salts, mixtures, metal-containing compounds, entries lacking numerical IC_50_ values, duplicate records with lower pIC_50_ values, and compounds with ambiguous stereochemistry were removed.

To enhance structural diversity and minimize redundancy, pairwise Tanimoto similarity coefficients were computed using ECFP6 molecular fingerprints. Compounds exhibiting high similarity (Tanimoto coefficient ≥ 0.80) were removed from the datasets prior to model development [4, 36]. The similarity coefficient distributions and the applied threshold are depicted in Fig. 1, where panel A corresponds to the AChE dataset and panel B to the BACE1 dataset.

Following curation, the AChE dataset was reduced from 6642 to 5340 unique compounds, while the BACE1 dataset was reduced from 7459 to 6116. All IC_50_ values were subsequently transformed into negative base-10 logarithmic units (pIC_50_) according to Eq. (1) to normalize the distribution and improve statistical model performance [33]:

1 $$ {\mathrm{pIC}}_{50} = - {\mathrm{log}}_{10} { }\left( {{\mathrm{IC}}_{50} \frac{{\left( {{\mathrm{mol}}/{\mathrm{L}}} \right)}}{1}} \right) $$

A detailed summary of record counts at each curation step for both AChE and BACE1 datasets is provided in Table 1.

**Table 1:** Summary of dataset curation steps for AChE and BACE1 bioactivity data

Target	AChE (n)	BACE1 (n)
Raw data	9731	10,764
After removal of missing IC_50_ values and ambiguous stereochemistry	8371	10,608
After removal of salts/mixtures/metals	6642	7549
After Tanimoto ≥ 0.80 redundancy filtering	5340	6116
Final curated dataset	5340	6116

### Dual inhibitor classification dataset construction

To construct the dual-inhibitor classification dataset, curated AChE and BACE1 bioactivity records were cross-matched to identify compounds with the same ChEMBL compound ID present in both datasets. This intersection yielded 204 compounds for which experimental activity data were available for both targets. All IC_50_ values were converted to pIC_50_ as described in Sect. “Dataset Acquisition and Preprocessing”.

Compounds were categorized into two classes based on predefined bioactivity thresholds [29]. Molecules with pIC_50_ values greater than 6.0 for both AChE and BACE1 were labeled as dual inhibitors (class = 1). If the pIC_50_ value for either target was ≤ 6.0, the compound was labeled as a non-dual inhibitor (class = 0). This threshold corresponds to an IC_50_ of 1 μM, a commonly accepted cut-off for high-affinity inhibition in medicinal chemistry [26]. In addition to the potency threshold used to define dual inhibitors (pIC_50_ > 6.0 for both AChE and BACE1), we further examined the consistency of bioactivity values within this subset. Among the 55 dual inhibitors, 31 compounds (56%) exhibited a pIC_50_ difference of less than 0.5 units between the two targets, which is within the commonly accepted experimental variability range for biochemical assays. This finding indicates that more than half of the dual inhibitors display highly comparable potency toward both AChE and BACE1, supporting the robustness and biological coherence of their dual-activity annotation. To assess the structural diversity of the dual inhibitor set, we calculated pairwise Tanimoto similarities (ECFP6) among 55 AChE/BACE1 dual inhibitors. In 1,485 pairwise comparisons, the mean Tanimoto similarity was 0.15 and the median was 0.10, indicating an overall low level of structural redundancy. Only 21 pairs (1.41%) showed a Tanimoto similarity greater than 0.7, and the highest observed similarity was 0.78, demonstrating that the dual inhibitor subset is chemically diverse. A similar trend was observed for the non-dual inhibitor class, with a mean Tanimoto similarity of 0.13, a median of 0.08, and only 111 out of 11,026 pairwise comparisons (1.01%) exceeding a similarity of 0.7. These results collectively confirm that both classes exhibit high chemical diversity, with only a small fraction of structurally similar compounds.

t-SNE is a statistical clustering method that visualizes high-dimensional data by assigning a location to each point on a two- or three-dimensional map [25]. As shown in Fig. 2, we used the t-distributed stochastic neighbor embedding (t-SNE) method to determine the statistical characteristics of all 204 compounds. To assess the chemical diversity and class overlap within the dataset, a t-SNE projection of ECFP6 fingerprints was generated for all 204 compounds. The resulting 2D chemical space showed partial overlap between dual (red) and non-dual (blue) inhibitors, indicating that the two classes occupy shared regions of the chemical landscape. This overlap suggests that dual inhibitors are not confined to a specific structural family and that the classification task is intrinsically challenging. The absence of distinct clustering further supports the structural heterogeneity of the dataset and highlights the need for a multi-metric and multi-model evaluation strategy.

Following classification, the dataset comprised 55 dual inhibitors and 149 non-dual inhibitors, reflecting a pronounced class imbalance. Table 2 summarizes the final class distribution along with descriptive statistics of AChE and BACE1 activities for each class. Strategies used to address this imbalance during model training are described in Sect. “Handling class imbalance”.

**Table 2:** Class distribution and summary statistics of dual inhibitors (Class 1) and non-dual inhibitors (Class 0) derived from the curated AChE and BACE1 bioactivity datasets. pIC_50_ values represent − log₁₀(IC_50_ in molar units)

Metric	Dual inhibitor (Class 1)	Non-dual inhibitor (Class 0)
Definition	pIC_50_ > 6.0 for both AChE and BACE1	pIC_50_ ≤ 6.0 for either target
No. of Compounds	55	149
Percentage (%)	26.96	73.04
AChE Mean pIC_50_ ± SD	7.38 ± 0.93	5.71 ± 1.36
AChE Range	6.05–9.72	1.54–10.05
BACE1 Mean pIC_50_ ± SD	7.21 ± 0.95	5.37 ± 0.75
BACE1 Range	6.00–9.32	3.30–7.00

### Regression dataset

Separate regression datasets were constructed for AChE and BACE1 to quantitatively predict inhibitory potency (pIC_50_) for each target. These datasets, derived from the curated bioactivity records described in Sect. “Dataset acquisition and preprocessing”, comprised 5340 unique compounds for AChE and 6116 for BACE1. Each dataset contained experimentally determined IC_50_ values converted to pIC_50_ units to serve as the dependent variable in regression modeling.

### Descriptor generation

To numerically encode the structural and physicochemical characteristics of the compounds, molecular descriptors and fingerprints were calculated. One-dimensional (1D) and two-dimensional (2D) descriptors were generated using the Mordred descriptor generator [34], which computes over 1,800 features covering constitutional, topological, and physicochemical properties. In addition, four fingerprint types were used to capture substructural patterns and atom connectivity: MACCS keys (166-bit predefined structural keys; [31]), Extended Connectivity Fingerprints with a diameter of 6 (ECFP6 [40]), PubChem fingerprints (881-bit predefined keys), and RDKit topological fingerprints (1024-bit path-based keys; [31]).

Descriptor and fingerprint generation was performed using Python (version 3.7) with the RDKit and Mordred libraries. These feature sets served as the basis for the subsequent feature selection procedures and machine learning model development described in the following sections. A complete summary of descriptor and fingerprint types, their dimensionality, and descriptions is provided in Table 3.

**Table 3:** Summary of descriptor and fingerprint families used for AChE and BACE1 modeling, including dimensionality, description, and software reference

Descriptor/Fingerprint type	No. of features	Description	Software/Referenceds
Mordred 1D Descriptors	1800 +	Physicochemical and constitutional properties (e.g., molecular weight, logP, atom counts)	Mordred [34]
Mordred 2D Descriptors	1800 +	Topological, connectivity, and electronic descriptors	Mordred [34]
MACCS Keys	166 bits	Predefined substructural keys	RDKit
ECFP6	2048 bits	Extended connectivity fingerprints, (radius = 3, diameter = 6)	RDKit
PubChem Fingerprints	881 bits	Predefined structural and functional group patterns	RDKit
RDKit Topological Fingerprints	1024 bits	Path-based fingerprints encoding atom connectivity	RDKit

Importantly, Mordred descriptors played a role only in the classification workflow. They were included in the 204-compound dual-inhibitor dataset for feature-selection benchmarking but were not used in the regression models. All similarity visualizations and interpretability analyses were based exclusively on fingerprint representations, primarily ECFP6, which yielded the highest classification performance.

### Feature selection

To reduce dimensionality and retain only the most informative variables, a multi-stage feature selection process was implemented exclusively on the 204-compound dual-inhibitor classification dataset to avoid data leakage. First, the Mutual Information (MI) criterion was applied to quantify the dependency between each descriptor and the target variable, and the top 200 descriptors from each descriptor set were retained [49, 53].

In the second stage, RF-based Recursive Feature Elimination (RF-RFE) and the Boruta algorithm were applied independently, each operating on the MI-reduced feature sets. RF-RFE iteratively removed the least informative features using Random Forest importance scores until 50 descriptors remained [19, 53]). Boruta, in contrast, evaluated feature relevance by comparing importance scores against randomized shadow features, retaining only statistically significant predictors [30]. Thus, the two algorithms were compared rather than combined, and each produced its own 50-feature subset used to train the classification models.

Within the regression workflow, no feature selection was applied. Instead, two fingerprint types—MACCS keys and ECFP6—were combined to assess whether structural (MACCS) and topological (ECFP6) information improved predictive performance. This combined representation was implemented only in the regression models, consistent with prior studies demonstrating complementary chemical information between these fingerprints [51].

### Machine learning models

Multiple supervised machine learning (ML) algorithms were evaluated to develop predictive classification models for identifying dual AChE/BACE1 inhibitors. The algorithms included *k*-Nearest Neighbors (KNN), Support Vector Machines (SVM), Random Forest (RF), Gradient Boosting Decision Trees (GBDT), Light Gradient Boosting Machine (LGBM), and Extreme Gradient Boosting (XGBoost). All models were implemented in Python (version 3.7) using the scikit-learn and LightGBM packages, with hyperparameters optimized via grid search combined with fivefold cross-validation [9, 28, 37, 42].

Model performance was assessed using multiple evaluation metrics, including accuracy, precision, recall, F1-score, Matthews Correlation Coefficient (MCC), area under the receiver operating characteristic curve (ROC-AUC), and area under the precision-recall curve (PR-AUC). In this study, the primary objective in evaluating classification performance was to evaluate the imbalanced nature of the dataset and preserve the biologically critical positive class (active compounds). Therefore, a hierarchical approach was employed for metrics. Initially, sensitivity (Recall) for the positive class was considered the primary criterion, and models with the highest Recall values ​​were selected. Among the models that met this requirement, the primary selection criteria were PR-AUC, which reflects the behavior of the minority class more accurately; F1 score, which summarizes the balance between precision and recall at a given decision threshold; and MCC, which demonstrates balanced discrimination between both classes. This holistic approach ensured both the complete capture of active compounds (minimizing false negatives) and the model\s overall classification performance at a balanced and reliable level.

Given the imbalanced nature of the dataset (55 dual inhibitors vs. 149 non-dual inhibitors), class weights were set to “balanced” in all models to ensure equal emphasis on both classes. Furthermore, the SMOTETomek algorithm was applied to oversample the minority class while simultaneously removing borderline samples from the majority class, thereby improving class balance [8, 44, 45].

The highest-performing models for each feature selection method (RF-based RFE and Boruta) and descriptor/fingerprint family are reported in the Results section, along with a comparative heatmap showing the metric distribution across models and feature types.

### Model evaluation metrics

Model performance was assessed using distinct evaluation criteria for classification and regression tasks.

For classification models, the following metrics were calculated:

- *Accuracy*—proportion of correct predictions among all predictions.
- *Precision*—proportion of correctly predicted positive cases among all predicted positives.
- *Recall (Sensitivity)*—proportion of actual positives correctly predicted by the model.
- *F1-score*—harmonic mean of precision and recall.
- *Matthews correlation coefficient (MCC)*—balanced measure accounting for true and false positives and negatives.
- *Area under the receiver operating characteristic curve (ROC-AUC)*—discrimination capacity across all thresholds.
- *Area under the precision–recall curve (PR-AUC)*—performance measure focusing on the positive class.

Additionally, a weighted performance score was calculated to facilitate model ranking by integrating multiple metrics into a single value. The weights assigned were: F1-score = 0.4, PR-AUC = 0.3, MCC = 0.2, and recall = 0.1. This composite measure allowed balanced comparison across different descriptor–algorithm combinations.

For regression models, performance was evaluated using:

- *Coefficient of determination (R*^*2*^*)*—proportion of variance explained by the model.
- *Cross-validated coefficient of determination (Q*^*2*^*)*—coefficient of determination computed under internal cross-validation, used to assess the internal predictive ability and robustness of the model.
- *Root mean squared error (RMSE)*—measure of prediction error magnitude.
- *Mean absolute error (MAE)*—average magnitude of prediction errors.

All evaluation metrics were computed using the scikit-learn package [37].

### External validation strategy

To rigorously evaluate the generalizability of the model, two independent external validation sets derived from experimentally measured activity values ​​in the ChEMBL 36 database were created. The first set consisted of 20 compounds selected to test the performance of the dual inhibitor classification model. The second set included 170 compounds with experimental pIC_50_C_50_ values ​​for both enzymes, which were used to evaluate the quantitative pIC_50_ prediction performance of AChE and BACE1 regression models. Both external sets were intentionally excluded from the model building, feature selection, and cross-validation steps to prevent potential data leakage. The GBDT–ECFP6 classifier, which demonstrated the best performance during the internal validation process, was applied to the external classification set, and classification metrics such as accuracy, precision, recall, F1-score, ROC-AUC, PR-AUC, and MCC were calculated. On the external set used for regression, AChE and BACE1 models were evaluated separately and standard regression metrics such as R^2^_ext, MAE_ext and MSE_ext were reported for external validation.

### Handling class imbalance

The dual-inhibitor classification dataset exhibited a pronounced class imbalance, with 55 dual inhibitors (class = 1) and 149 non-dual inhibitors (class = 0), corresponding to a 1:2.7 ratio (see Sect. “Dual Inhibitor Classification Dataset Construction”). Such imbalance can bias the learning process toward the majority class, reducing the model’s ability to correctly identify minority-class compounds of interest.

To address this, two complementary strategies were employed:

1. *Class weight adjustment*—All classification models were trained with class_weight = ”balanced”, which scales the contribution of each class inversely proportional to its frequency, ensuring that minority-class samples have greater influence during model fitting.
2. *SMOTETomek resampling*—The Synthetic Minority Over-sampling Technique combined with Tomek links (SMOTETomek) was applied to further improve class balance [8, 45]. This hybrid method oversamples the minority class by generating synthetic samples along the line segments joining minority-class instances and their nearest neighbors, while simultaneously removing borderline majority-class instances identified by Tomek links. This process enhances the separation between classes and reduces the likelihood of overlapping decision boundaries.

The combination of class weighting and SMOTETomek resampling provided a balanced training distribution while preserving chemically meaningful structural diversity in both classes.

#### Model explainability (SHAP analysis)

To interpret the contribution of individual molecular features to model predictions, SHapley Additive exPlanations (SHAP) analysis was employed [32]. SHAP values, derived from cooperative game theory, quantify the marginal contribution of each feature to the predicted output, thereby enabling the decomposition of a model’s prediction into additive feature contributions. This approach was applied to both classification and regression models to identify substructural elements that positively or negatively influence dual AChE/BACE1 inhibition.

For fingerprint-based models, SHAP values were calculated for each bit position, allowing direct association of specific substructures—such as aromatic rings, halogen substituents, alkyl chains, or heterocyclic motifs—with enhanced or reduced inhibitory activity. This interpretation facilitated the elucidation of structure–activity relationships (SAR), supporting the rational design of novel dual inhibitors.

SHAP analyses were performed using the Python SHAP package (version 0.44.1), applying **TreeExplainer** for tree-based models (GBDT, RF, XGBoost, LGBM) and **KernelExplainer** for SVM models. For each model, SHAP value outputs were visualized as feature importance bar plots and global summary plots (beeswarm format), along with molecular fragment depictions for the most influential fingerprint bits.

For the best-performing classifier (GBDT–ECFP6), the top-ranked bits by mean |SHAP| values and their associated substructures are presented in Supplementary Table S1, with corresponding bar and summary plots shown in Figs. 7 and 8. Positive SHAP values indicate features that increase the predicted probability of dual inhibition, whereas negative values indicate features that decrease it.

The results of the most successful regression models developed for the AChE and BACE1 enzymes are presented in Table 5 of the Results section. Summary plots and bar graphs associated with these models are visualized in Figs. 9 (AChE) and 10 (BACE1), respectively. According to the SHAP analysis, positive SHAP values ​​represent features that increase the predicted pIC_50_ value of the respective compound on AChE and BACE1, while negative SHAP values ​​represent molecular features that decrease this value.

#### Applicability domain (AD) analysis

To assess the chemical space coverage and reliability of model predictions, an Applicability Domain (AD) analysis was conducted. The AD defines the region of chemical space within which a (Q)SAR model can make reliable predictions with a defined level of confidence [15, 46, 47]. In this study, the AD was evaluated using the **Williams plot**, which relates the standardized residuals of model predictions to their corresponding leverage (h) values [16].

The **leverage** quantifies the influence of each compound on the model, with higher values indicating stronger influence. The threshold leverage (h*) was calculated according to formula:

$$ h^{*} = \frac{{3\left( {p + 1} \right)}}{n} $$

where *p* is the number of model descriptors and *n* is the number of compounds in the training set. Compounds with leverage values greater than h* or with standardized residuals exceeding ± 3 standard deviations were considered **outliers** and classified as lying outside the AD.

The Williams plot provides a visual representation of this analysis, with the horizontal boundaries at standardized residuals =  ± 3 and the vertical boundary at h* demarcating the AD limits. AD assessments were performed regression models, with plots generated using Python’s Matplotlib and Seaborn libraries.

The AD results, including the proportion of compounds falling within the domain for both training and test sets, are presented in the Results section (Fig. 11).

#### ADMET predictions and molecular docking experiments

The ChEMBL accession numbers of the ligands predicted as potential dual AChE/BACE1 inhibitors through SHAP analysis were retrieved. For ADMET prediction, the SMILES format representations of these ligands were obtained from the ChEMBL database. Their three-dimensional (3D) structural information was also downloaded in MDL Molfile format. Hydrogen atoms were subsequently added, and energy minimization was carried out using the MMFF94 force field implemented in Avogadro 1.2.0 [23]. The optimized ligand structures were then exported in mol2 format for use in molecular docking studies.

The SwissADME web tool (http://www.swissadme.ch) was utilized to evaluate the bioavailability and pharmacokinetic properties of the ligands, as well as to predict their overall drug-likeness. In this context, the ligands were assessed for compliance with Lipinski’s Rule of Five (LogP < 5, H-bond acceptors < 10, MW < 500 Da, H-bond donors < 5).

Toxicity profiling was conducted using the pkCSM web server (https://biosig.lab.uq.edu.au/pkcsm/prediction), where the ligands were systematically evaluated for blood–brain barrier (BBB) permeability, AMES mutagenesis, liver injury I, liver injury II, and hERG blockade.

Molecular docking simulations were conducted to evaluate the binding modes and affinities of the top-predicted high-confidence dual AChE/BACE1 inhibitors. Crystal structures of AChE (PDB ID: 4EY7, 2.35 Å; LBI value: 0.972) and BACE1 (PDB ID: 2G94, 1.86 Å; LBI value: 0.998) were retrieved from the Protein Data Bank. These structures were prioritized based on several structural-quality indicators, including high crystallographic resolution, the absence of missing residues in the active site, the presence of suitable co-crystallized ligands, and Ligand B-factor Index (LBI) values within the recommended 0.8–1.2 range [21]. All crystallographic water molecules, ions, and co-crystallized ligands were removed, except for those essential for structural stability or catalytic function, as determined by literature reports and binding site conservation analysis. Polar hydrogens were added, and Kollman charges were assigned using **AutoDockTools v1.5.6** [35].

The minimized ligands were assigned torsional flexibility using AutoDockTools. The docking grid box was centered on the binding site of the co-crystallized inhibitor ligand for each protein. Grid box parameters were defined with the AGFR 1.2 program. For AChE, the grid box was set to encompass the entire active gorge (center: − 13.988, − 43.906, 27.108; size: 60 × 40 × 45 Å), whereas for BACE1 it was adjusted to cover the catalytic cleft (center: -− 4.329, − 4.043, 30.651; size: 60 × 40 × 40 Å). Docking simulations were performed using AutoDock 4.2.6 with the Lamarckian Genetic Algorithm (LGA). The parameters applied were as follows: population size = 150, maximum number of energy evaluations = 2.5 × 10^6^, maximum number of generations = 27,000, and 200 independent docking runs per ligand.

Binding affinities were reported as predicted binding free energies (ΔG, kcal·mol^−1^) calculated using the AutoDock scoring function. Docking poses were ranked according to binding energy, and the top-ranked pose of each ligand was subjected to interaction analysis with BIOVIA Discovery Studio Visualizer v25.1.0 [13]. Interaction profiling encompassed hydrogen bonding, π–π stacking, π–cation interactions, hydrophobic contacts, and van der Waals forces.

Docking validation was performed via **redocking** of the original co-crystallized ligands (donepezil for AChE, CHEMBL448008 for BACE1) into their respective binding sites. RMSD values between experimental and redocked poses were calculated to ensure docking accuracy, with RMSD < 2.0 Å considered acceptable. Pearson’s correlation analysis and correlation plot generation were performed in R (version 4.5.2) to evaluate the methodological consistency of the docking protocol, using ligand datasets specific to AChE and BACE1.

## Results and discussion

### Classification model performance and interpretation

**Fig. 4:**
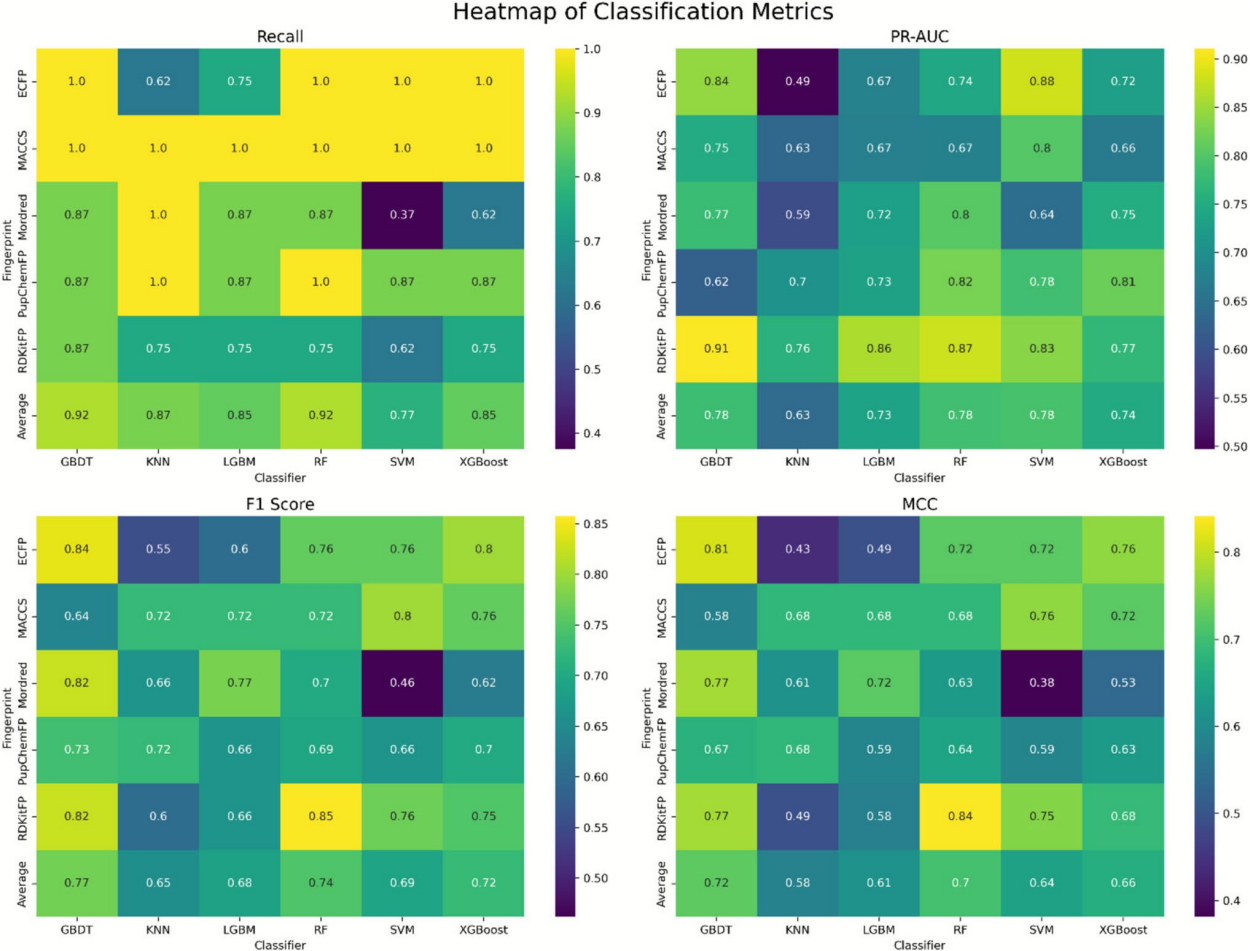
Heatmap of Classification Metrics for six classifiers. Comparative performance of GBDT, KNN, LGBM, RF, SVM and XGBoost across multiple descriptor and fingerprint types. Metrics that provide a balanced evaluation of classification performance (Recall, PR-AUC, F1-score, MCC)

The classification performance of the developed machine learning models was systematically assessed across multiple molecular descriptor and fingerprint types. As illustrated in the heatmap of classification metrics (Fig. 4), GBDT model trained on ECFP6 fingerprints achieved the highest performance among all other classifier-descriptors. Notably, Random Forest (RF) with ECFP6 and Support Vector Machine with ECFP6 ranked closely behind, consistent with previous reports demonstrating the high discriminatory capacity of extended connectivity fingerprints in cheminformatics classification tasks [4, 39].

A detailed comparison of the top five classification models is provided in Table 4**.** The GBDT–ECFP6 model exhibited a balanced and superior performance profile, with **Recall (1.00)**, PR-AUC (**0.84**), **F1-score (0.84)** and MCC (0.81) values. The perfect recall is particularly advantageous for early-phase virtual screening, where minimizing false negatives is critical to avoid discarding potentially active compounds [24].

**Table 4:** Performance metrics of the top five classification models for dual AChE–BACE1 inhibition prediction using Random Forest and Boruta feature sets

	Fingerprint	Accuracy	Precision	Recall	F1 Score	PR-AUC	AUC-ROC	MCC	Model
Random Forest	**ECFP6**	**0.92**	**0.72**	**1.00**	**0.84**	**0.86**	**0.96**	**0.81**	**GBDT**
	RDkit-FP	0.95	1.00	0.75	0.85	0.87	0.92	0.84	RF
	RDkit-FP	0.92	0.77	0.87	0.82	0.91	0.92	0.77	GBDT
	MACCS	0.9	0.66	1.00	0.8	0.8	0.96	0.76	SVM
	ECFP6	0.87	0.61	1.00	0.76	0.88	0.96	0.72	SVM
Boruta	**MACCS**	**0.9**	**0.66**	**1**	**0.8**	**0.87**	**0.96**	**0.76**	**XGBoost**
	MACCS	0.87	0.61	1	0.76	0.87	0.96	0.72	RF
	RDkit-FP	0.92	0.85	0.75	0.8	0.86	0.92	0.75	RF
	ECFP6	0.9	0.66	1	0.8	0.69	0.94	0.76	KNN
	RDkit-FP	0.92	1	0.62	0.76	0.83	0.89	0.75	SVM

Columns: Fingerprint, Accuracy, Precision, Recall, F1-score, PR-AUC, ROC-AUC, MCC, Model

Bold values represent the best-performing models within each feature selection approach

**Fig. 5:**
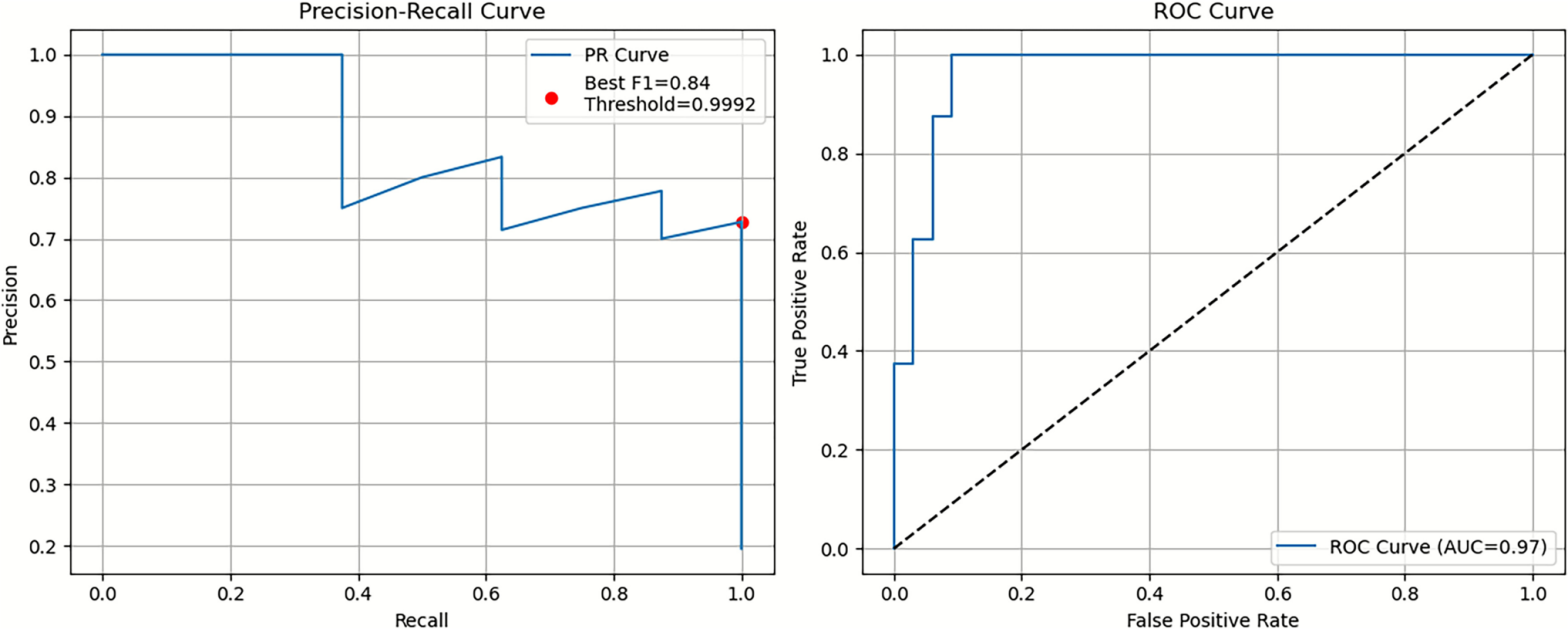
Precision–Recall and ROC curves of the GBDT–ECFP6 model. Performance evaluation of the Gradient Boosting Decision Tree (GBDT) classifier trained on ECFP6 fingerprints. Panel A shows the Precision–Recall curve, where the best F1 score (0.84) is marked with a red dot and the corresponding threshold (0.9992) is indicated. Panel B depicts the Receiver Operating Characteristic (ROC) curve with an area under the curve (AUC) of 0.97. The dashed diagonal line represents random classification performance

The model’s robustness was further confirmed by precision–recall (PR) and receiver operating characteristic (ROC) curve analyses (Fig. 5). The PR curve indicated a best F1-score of **0.84**, while the ROC curve yielded an AUC of **0.97**, exceeding the conventional 0.80 threshold for strong classification models in cheminformatics applications.

Importantly, when the same GBDT–ECFP6 classifier was challenged on an independent external validation set comprising 20 previously unseen compounds, it retained good predictive performance (Accuracy = 0.85, F1 score = 0.72, Recall = 0.80, PR-AUC = 0.88, MCC = 0.62). These external validation results indicate that the classifier generalizes reasonably well to novel chemotypes and that the high internal PR-AUC and ROC-AUC values are not merely a consequence of overfitting.

**Fig. 6:**
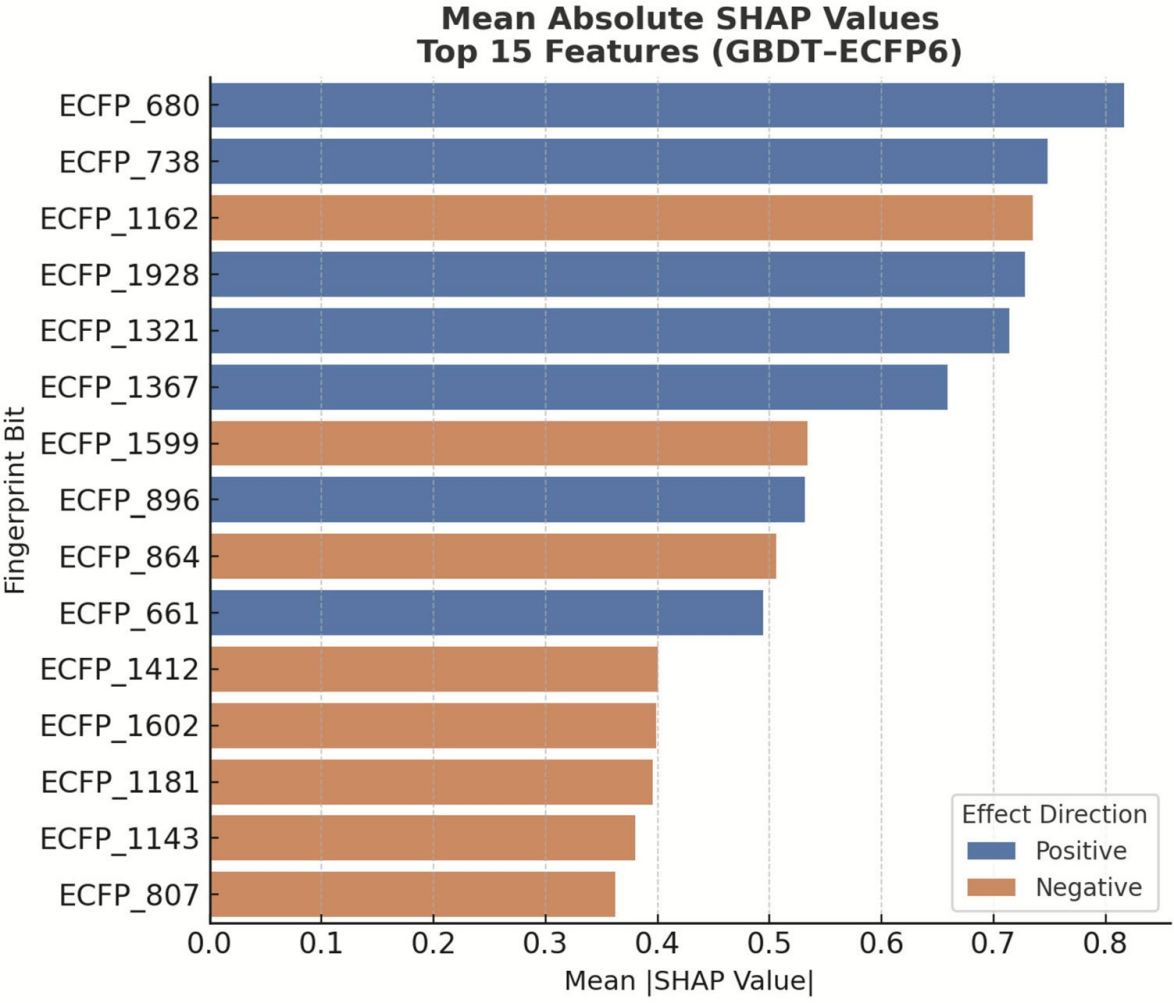
Top SHAP-ranked ECFP6 features in the classification model. Bar plot of mean absolute SHAP values for the 15 most influential ECFP6 fingerprint bits in the GBDT–ECFP6 classification model. Blue bars represent features positively associated with inhibition probability, while orange bars indicate features negatively associated with activity. Larger SHAP values reflect greater influence on the model output, providing structural insight into molecular determinants that drive classification decisions

**Fig. 7:**
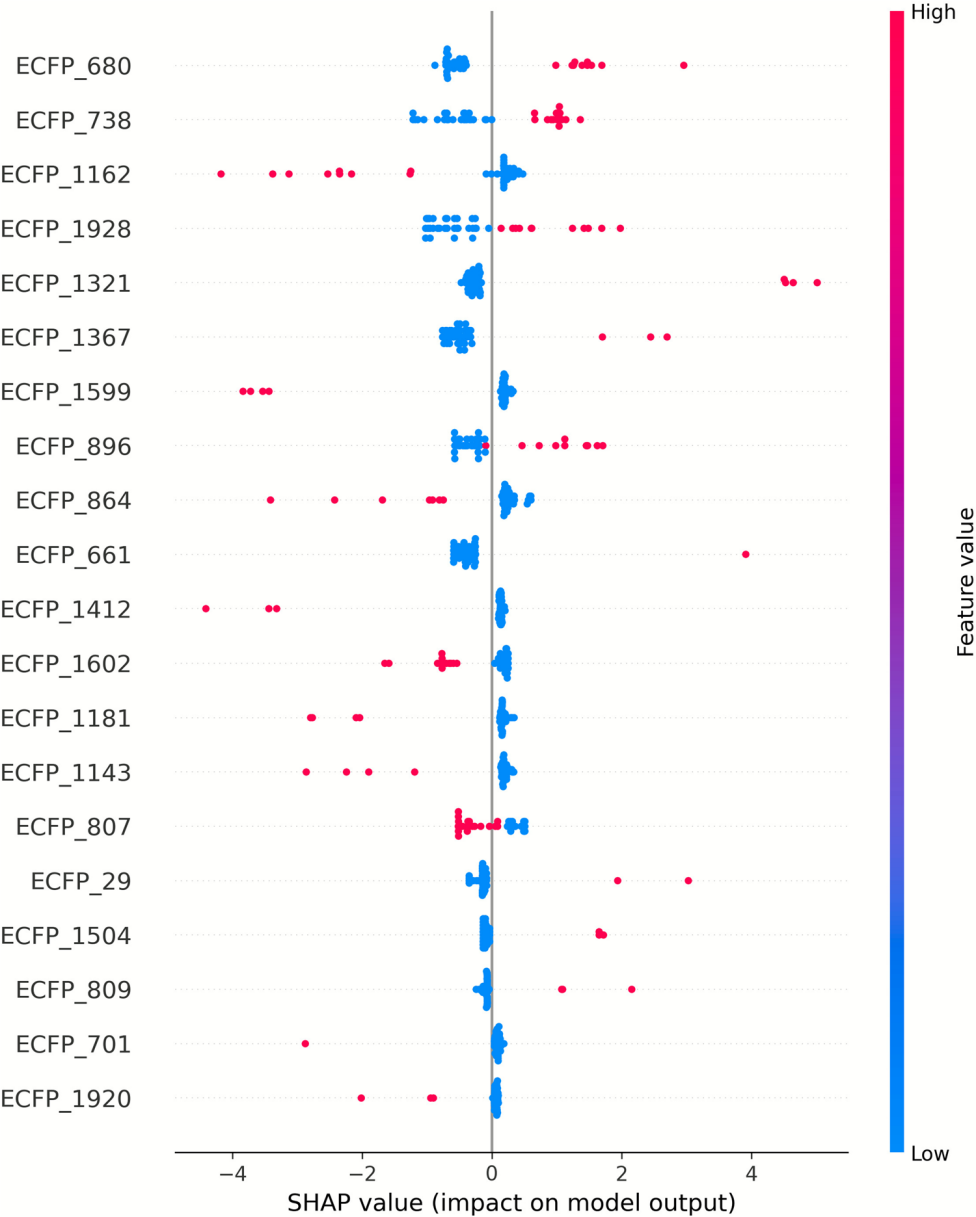
SHAP summary plot of top ECFP6 features. Beeswarm plot illustrating the top 20 ECFP6 fingerprint bits that contributed most to predictions in the GBDT–ECFP6 classification model. Each point represents a compound, positioned along the x-axis by its SHAP value (impact on the model output) and colored according to the normalized feature value (red = high, blue = low). Positive SHAP values indicate features driving predictions toward the dual-inhibitor class, while negative values indicate features favoring the non-dual inhibitor class

To provide interpretability, SHAP analysis was applied to identify the molecular features most strongly influencing classification outcomes. The top 15 ECFP6 fingerprint bits for the GBDT–ECFP6 model are listed in Fig. 6, with positive SHAP values indicating features associated with the dual-inhibitor class and negative SHAP values indicating features associated with the non-dual inhibitor class. The SHAP beeswarm plot (Fig. 7) highlighted several high-impact features, such as ECFP_680, ECFP_738, and ECFP_1321, which correspond to aromatic or heteroaromatic scaffolds known to enhance binding affinity to both cholinesterases and β-secretase [20, 54]. The corresponding numerical SHAP values are provided in Supplementary Table S1.

**Fig. 8:**
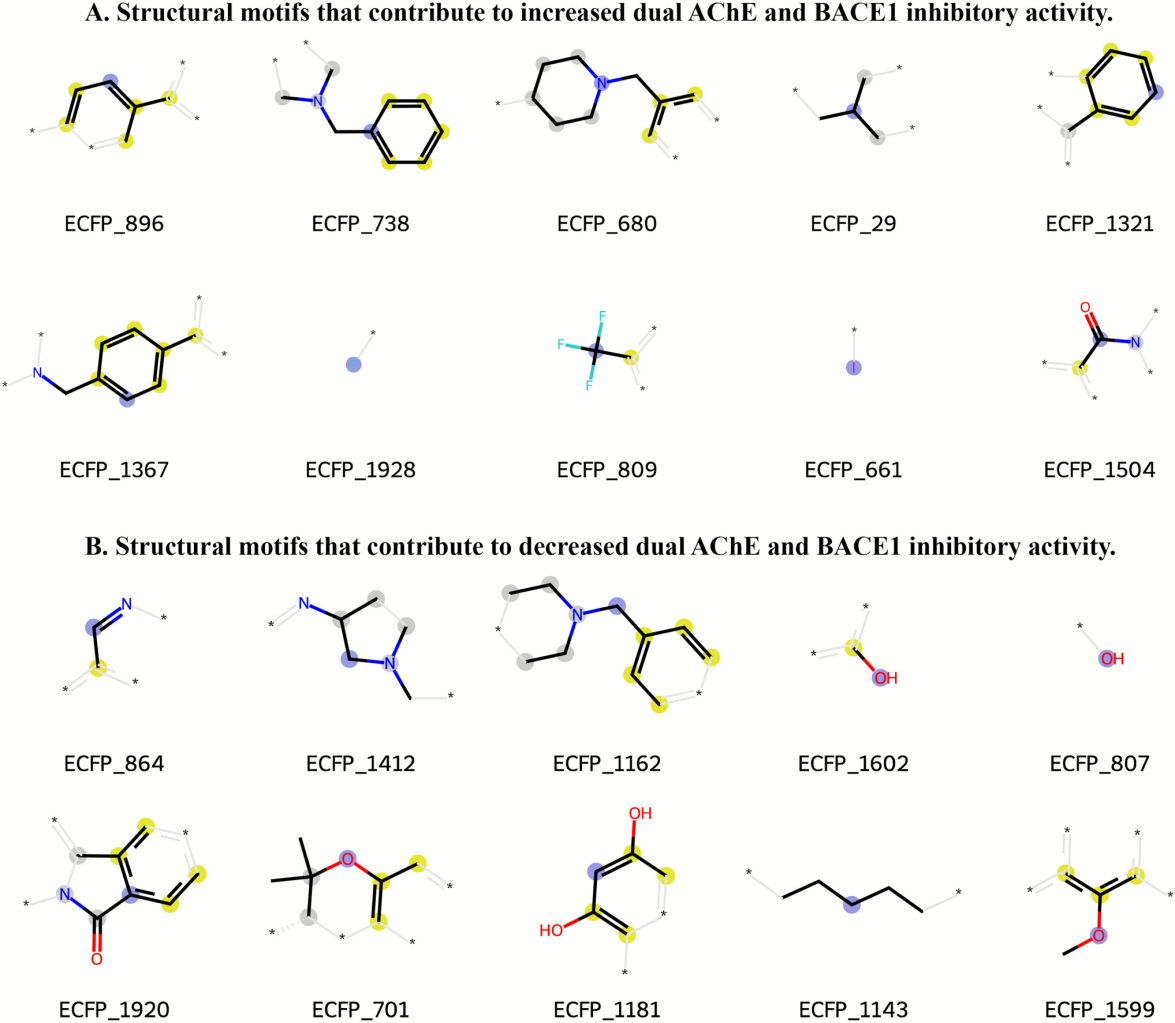
Representative substructures from SHAP-ranked ECFP6 features. Chemical fragments corresponding to the top-ranked ECFP6 fingerprint bits identified by SHAP analysis in the GBDT–ECFP6 classification model. Blue atoms indicate the central atom, gray atoms represent aliphatic ring atoms, and yellow atoms denote aromatic atoms. Panel A highlights motifs associated with increased probability of dual AChE–BACE1 inhibitory activity, whereas Panel B shows motifs linked to decreased probability

Representative chemical substructures corresponding to these influential bits are shown in Fig. 8. Blue atoms denote the central atom, yellow indicates aromatic atoms, and gray represents aliphatic ring atoms. Many of these motifs—such as fused aromatic systems and heterocycles containing nitrogen or oxygen—are known to facilitate π–π stacking, hydrogen bonding, and hydrophobic interactions, which are critical in stabilizing ligand binding within the active sites of AChE and BACE1.

Overall, these results demonstrate that the integration of gradient boosting with ECFP6 fingerprint representation enables accurate and interpretable classification of potential dual AChE/BACE1 inhibitors. The identification of chemically meaningful substructures through SHAP not only validates the model’s predictions but also provides a rational basis for subsequent molecular optimization and docking studies.

### Regression model performance and SHAP-based feature interpretation

The regression models developed for quantitative prediction of AChE and BACE1 inhibitory potency (pIC_50__50_) exhibited consistent and robust performance across multiple machine learning algorithms (Table 5). For AChE, the Support Vector Machine (SVM) model achieved the highest coefficient of determination (**R**^**2**^** = 0.697**) and the lowest mean squared error (**MSE = 0.624**), closely followed by the Gradient Boosting Decision Tree (GBDT) model (**R**^**2**^** = 0.692**, **MSE = 0.633**). For BACE1, the best predictive accuracy was also observed with the SVM model (**R**^**2**^** = 0.703, MSE = 0.582**), indicating its superior generalization capability for both targets. These R^2^ values (> 0.68) are comparable to or exceed those reported in recent QSAR studies targeting cholinesterases and secretases [10, 54], underscoring the reliability of the developed models. For AChE, the GBDT model provided external performance (R^2^_ext = 0.617, MAE_ext = 0.455, MSE_ext = 0.305) and was therefore selected as the final regression model. Similarly, for BACE1, although GBDT had only slightly lower internal R^2^ than SVM, it outperformed the other algorithms on the external test set (R^2^_ext = 0.654, MAE_ext = 0.573, MSE_ext = 0.488) and was selected as the optimal prediction model [47].

**Table 5:** Regression performance metrics for AChE and BACE1 prediction models

	Model	R^2^	Q^2^	MAE	MSE	R^2^_ext^*^	MAE_ext^*^	MSE_ext^*^
AChE	RF	0.677	0.678	0.567	0.664	0.494	0.516	0.403
	KNN	0.687	0.688	0.537	0.644	0.253	0.598	0.596
	LGBM	0.678	0.679	0.566	0.663	0.458	0.515	0.432
	GBDT	**0.695**	**0.695**	**0.551**	**0.628**	**0.617**	**0.455**	**0.305**
	SVM	0.697	0.698	0.555	0.624	0.547	0.483	0.361
	XGBoost	0.659	0.660	0.594	0.702	0.532	0.511	0.373
BACE1	RF	0.686	0.687	0.562	0.615	0.587	0.625	0.582
	KNN	0.678	0.679	0.565	0.631	0.454	0.662	0.770
	LGBM	0.692	0.693	0.570	0.602	0.634	0.581	0.515
	GBDT	**0.696**	**0.697**	**0.562**	**0.595**	**0.654**	**0.573**	**0.488**
	SVM	0.702	0.703	0.549	0.583	0.586	0.596	0.582
	XGBoost	0.687	0.688	0.581	0.613	0.593	0.616	0.573

The best performance values for each metric are highlighted in bold. Asterisked metrics (R^2^_ext, MAE_ext, MSE_ext) correspond to external validation on an independent test set

Bold values represent the best regression performance for each target, considering both internal and external validation metrics

**Fig. 9:**
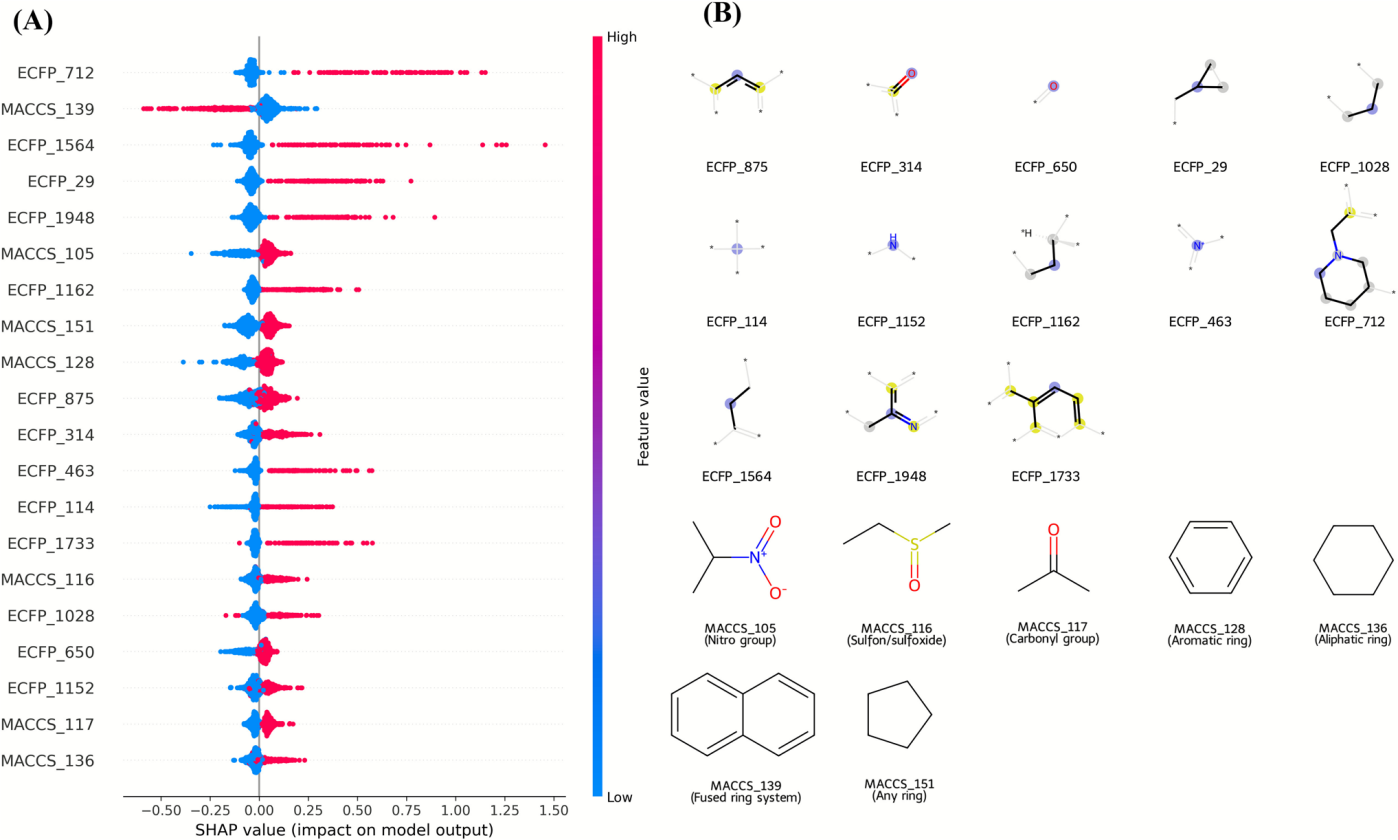
SHAP features and substructures in the AChE model. Summary plot and representative molecular fragments corresponding to the top ECFP6 and MACCS fingerprint features contributing to the AChE regression model. Panel A shows SHAP values illustrating the impact of each fingerprint on model predictions, with red indicating high feature values and blue indicating low values. Panel B depicts the molecular substructures associated with the most influential fingerprint bits, highlighting motifs that drive predicted inhibitory potency

SHAP-based feature interpretation revealed the molecular determinants influencing regression predictions for both enzymes. For AChE, the most impactful features comprised a combination of ECFP6 and MACCS fingerprint bits (Fig. 9A), with high positive SHAP values associated with aromatic systems, alicyclic scaffolds, and sulfonyl-containing moieties (Fig. 9B). Structural motifs such as naphthalene derivatives (MACCS_128) and heterocyclic and aromatic-related substructures (e.g., ECFP_29 and ECFP_712) were positively correlated with increased pIC_50_ values, consistent with literature reports highlighting aromatic stacking and hydrophobic interactions as key contributors to AChE inhibition [2, 3, 11, 20]. Conversely, certain aliphatic functionalities (e.g., MACCS_136, MACCS_116) exhibited negative SHAP effects, suggesting reduced potency.

**Fig. 10:**
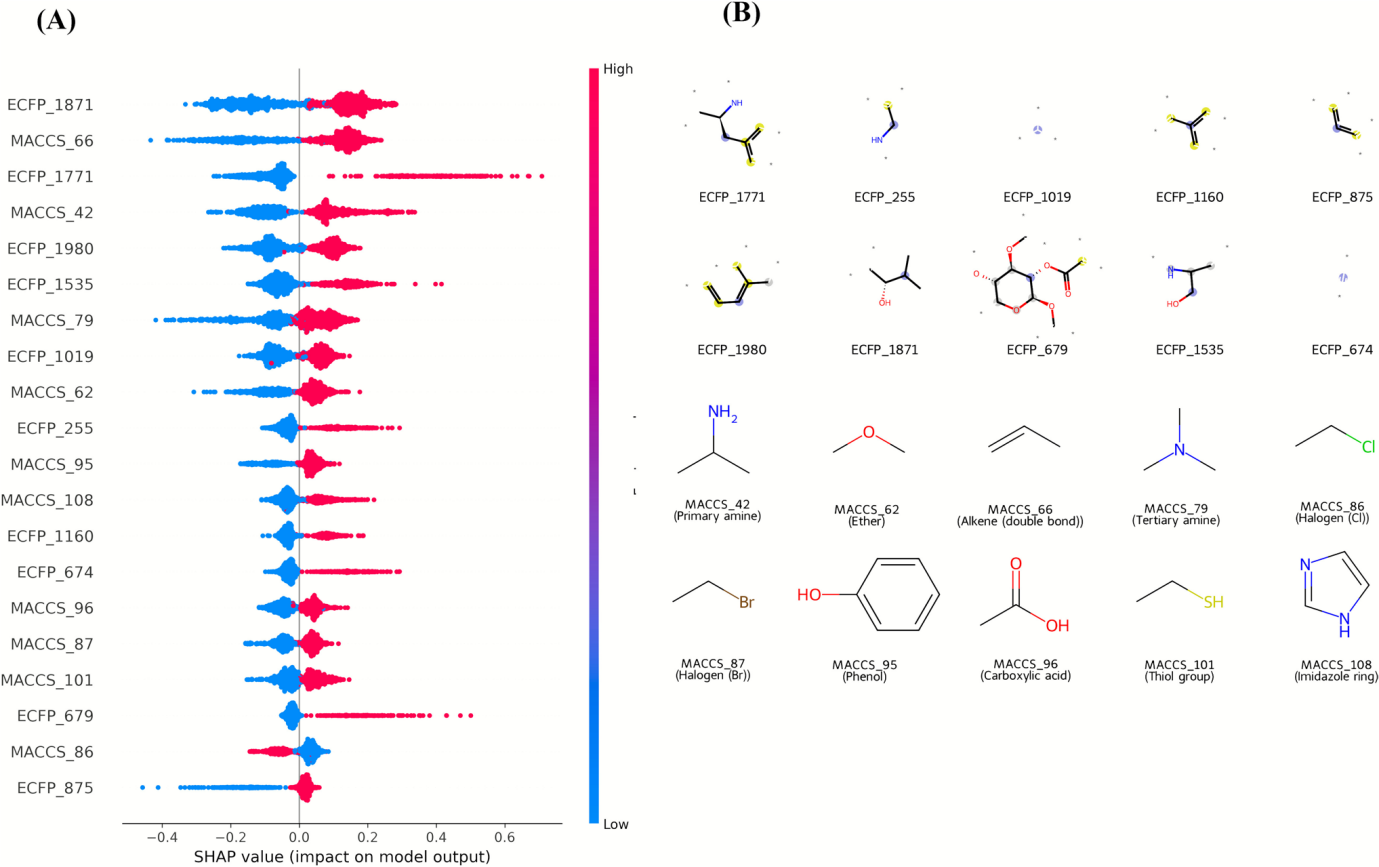
SHAP features and substructures in the BACE1 model. Summary plot and representative molecular fragments corresponding to the top ECFP6 and MACCS fingerprint features contributing to the BACE1 regression model. Panel A shows SHAP values illustrating the impact of each fingerprint on model predictions, with red indicating high feature values and blue indicating low values. Panel B depicts the molecular substructures associated with the most influential fingerprint bits, highlighting motifs linked to predicted inhibitory potency

For BACE1, SHAP analysis similarly indicated a dominance of aromatic and heteroaromatic moieties among positively contributing features (Fig. 10A). Representative substructures included substituted benzene rings, bicyclic aromatic heterocycles, and hydrogen bond–accepting groups such as ethers and amides (Fig. 10B). Notably, features like ECFP_1871 and ECFP_1771 were strongly associated with higher predicted potency, in agreement with prior molecular modeling studies that emphasized the role of extended aromatic scaffolds and polar substituents in enhancing BACE1 binding affinity [5, 18, 52]. Negative SHAP contributions were primarily linked to small alkyl chains and halogen-substituted aliphatics, indicating that overly simple hydrophobic groups may be less favorable for dual-target binding.

Collectively, the regression performance metrics and SHAP-derived structural insights confirm that the developed models not only achieve high predictive accuracy but also capture chemically interpretable structure–activity relationships. This interpretability provides a rational foundation for prioritizing compounds with desirable substructural motifs in subsequent design and molecular docking stages.

### Applicability domain and high-confidence dual inhibitor selection

**Fig. 11:**
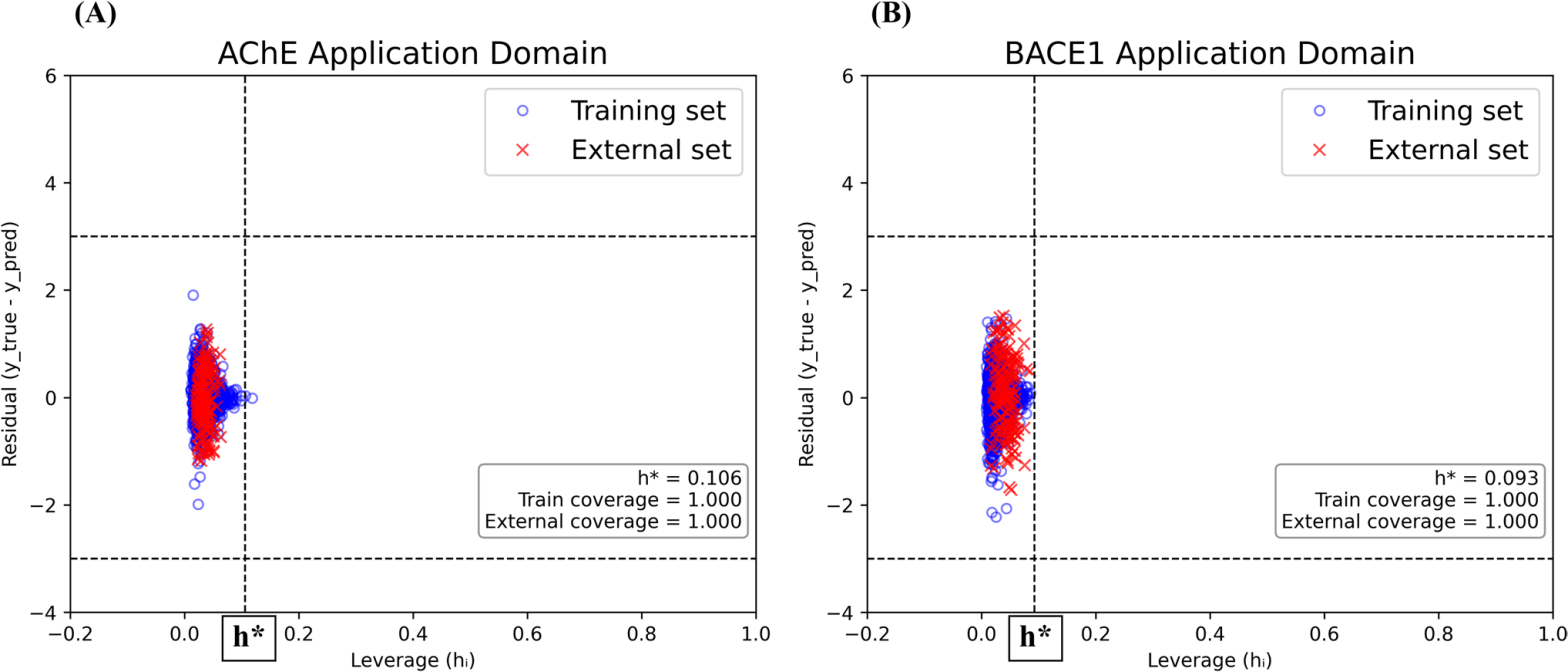
Williams plots of applicability domain for regression models. Applicability domain (AD) analysis of the regression models for acetylcholinesterase (AChE,** A**) and β-secretase 1 (BACE1,** B**). The x-axis shows leverage (h), and the y-axis shows standardized residuals. The vertical dashed line marks the leverage threshold (h⁎), while the horizontal dashed lines correspond to ± 3 standardized residuals. Blue circles represent training set compounds, and red crosses indicate test set compounds. Most data points fall within the AD boundaries, confirming broad model coverage and reliable predictive performance

The robustness and reliability of the regression models were assessed through Applicability Domain (AD) analysis using Williams plots (Fig. 11). For each target, the AD was defined on the basis of the training set, and the compounds from the independent external validation set (n = 170) were subsequently projected into this chemical space. In both the AChE and BACE1 regression models, more than 99,9% of the training compounds and all external validation compounds fell within the defined leverage thresholds (h⁎ = 0.106 for AChE; h⁎ = 0.093 for BACE1) and within the ± 3 standardized residual limits, indicating broad model coverage. This result confirms that the vast majority of predictions, including those for the external set, were made within well-represented regions of the models’ chemical space, thereby reducing the risk of extrapolative errors.

**Fig. 12:**
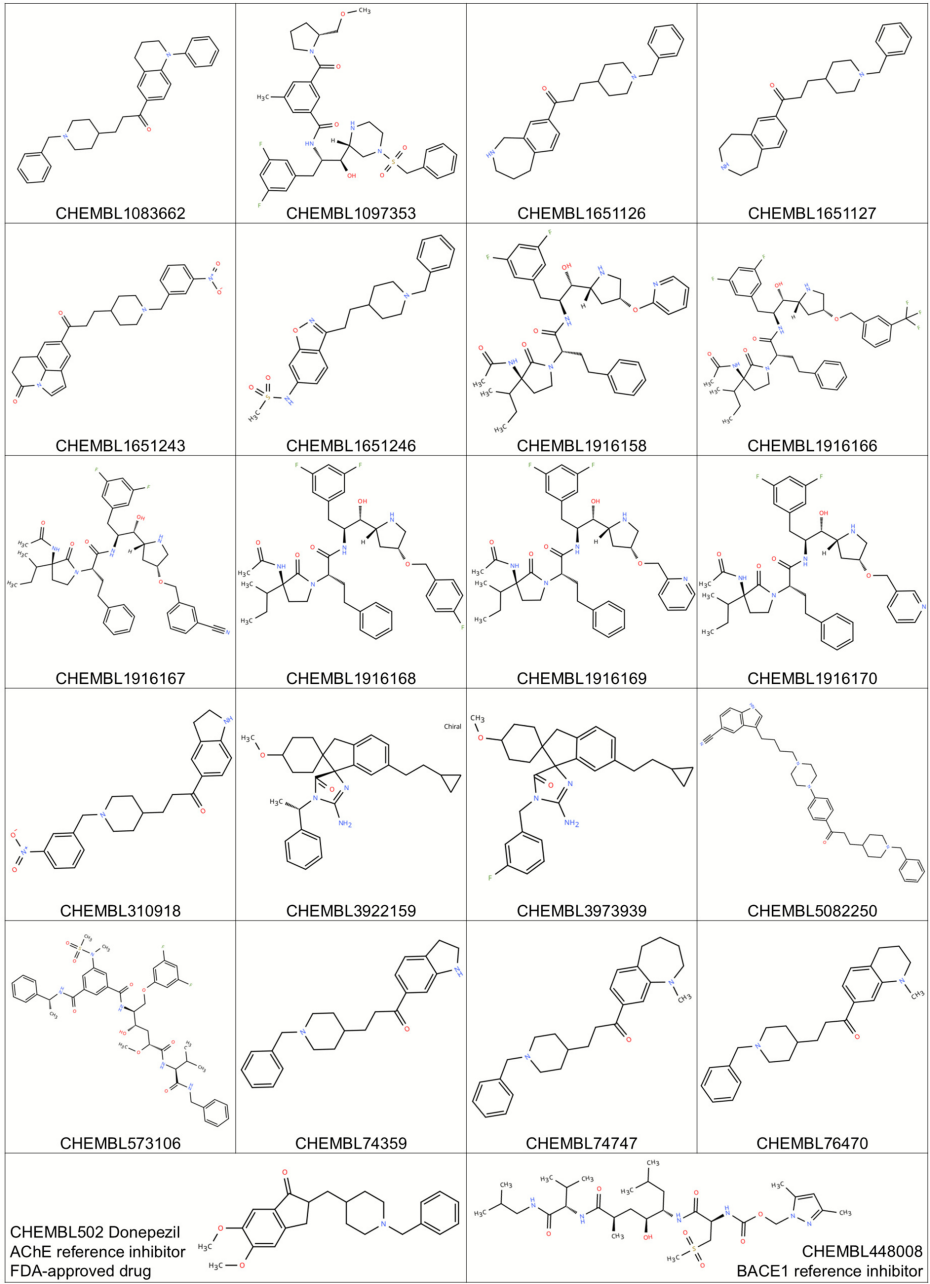
2D structural visualizations of high-confidence dual inhibitor candidates and reference inhibitors used in the molecular docking studies

First, the applicability domains (AD) of the AChE and BACE1 regression models were defined based on their respective training sets using Williams plots. For each model, the leverage threshold (h*) was calculated from the training descriptors, and this threshold was subsequently used as a structural filter to decide whether new compounds fall inside the reliable chemical space of the corresponding model.In the next step, we propagated information between the two regression datasets in a cross-target manner. Specifically, the AChE regression model was applied to the BACE1 regression dataset to obtain predicted AChE pIC_50_ values for BACE1 compounds, and the BACE1 regression model was applied to the AChE regression dataset to obtain predicted BACE1 pIC_50_ values for AChE compounds. For each of these cross-target predictions, the leverage was computed with respect to the training set of the corresponding model, and only compounds with h ≤ h* were retained as AD-compliant cross-target predictions. In parallel, the GBDT–ECFP6 classifier was applied to the same AChE and BACE1 regression datasets to estimate the probability that each compound is a dual inhibitor. Thus, for every compound lying inside the AD of both regression models, we obtained: (i) a dual-inhibitor probability from the classifier and (ii) predicted pIC_50_ values for both AChE and BACE1 from the respective regression models (including the cross-target predictions). Based on these AD-filtered predictions, a set of high-confidence dual inhibitor candidates was identified (Table 6). Compounds were selected if they fulfilled two criteria: (1) a predicted dual-inhibitor probability ≥ 0.999 in the GBDT–ECFP6 classifier, and (2) predicted pIC_50_ values for both AChE and BACE1 falling within the top 5% of the corresponding regression output distributions while remaining inside the applicability domain of both models. These high-confidence candidates were subsequently advanced to molecular docking studies to explore their binding modes and validate their dual-target potential (Table 6, Fig. 12).

**Table 6:** High-confidence dual inhibitor candidates for AChE and BACE1 were identified based on the outputs of the dual-inhibitor classification model and the regression models

BACE1 Molecule ID	BACE1 pIC_50_	Classification Probability (BACE1)	Predicted AChE pIC_50_	AChE Molecule ID	AChE pIC_50_	Classification Probability (AChE)	Predicted BACE1 pIC_50_
CHEMBL 1,097,353	7.85	0.99	6.85	CHEMBL 1,651,246	7.85	0.99	7.06
CHEMBL 1,916,166	8.57	0.99	7.09	CHEMBL 74,359	6.95	0.99	7.28
CHEMBL 1,916,158	8.59	0.99	6.87	CHEMBL 74,747	7.09	0.99	7.28
CHEMBL 1,916,168	8	0.99	7.14	CHEMBL 1,651,127	7.6	0.99	7.04
CHEMBL 1,916,170	8.85	0.99	6.87	CHEMBL 76,470	7.44	0.99	7.28
CHEMBL 1,916,169	8.82	0.99	6.87	CHEMBL 1,651,126	7.64	0.99	7.04
CHEMBL 1,916,167	8.52	0.99	6.81	CHEMBL 1,083,662	7.27	0.99	6.94
CHEMBL 3,973,939	7.8	0.99	7.2	CHEMBL 1,651,243	8.54	0.99	7.39
CHEMBL 573,106	9.46	0.99	7.17	CHEMBL 310,918	7.19	0.99	7.32
CHEMBL 3,922,159	7.01	0.99	7.41	CHEMBL 5082250	9.24	0.99	7.03

Compounds were selected if they (i) were classified as dual inhibitors with a predicted probability ≥ 0.999 in the GBDT–ECFP6 classifier, and (ii) had predicted pIC_50_ values ​​within the top 5% of the regression model outputs for both targets

### ADMET profiling and drug-likeness evaluation

ADMET evaluation provides a systematic assessment of pharmacokinetic behavior and toxicity profiles of candidate molecules, enabling early prediction of drug-likeness, efficacy, and safety. Such predictive profiling is critical to reducing late-stage attrition and enhancing the translational value of drug discovery [27]. In this study, ADMET properties were comprehensively analyzed using the widely adopted SwissADME and pkCSM platforms for the 20 ligands selected through the QSAR workflow (Table 6), and two reference inhibitors (CHEMBL502 and CHEMBL448008) were assessed for comparison. Representative ADMET properties of the most relevant ligands are summarized in Table 7, highlighting BBB permeability, mutagenicity, and hERG liability as the most critical determinants for translational prioritization. Full ADMET datasets for all compounds are provided in Supplementary Table S2.

**Table 7:** Summary of key ADMET properties of representative ligands

Compound ID	Lipinski violations	BBB permeability	AMES mutagenicity	hERG inhibition	Overall assessment
CHEMBL502 (Donepezil)	0	Yes	Non-mutagenic	Risk	Clinically approved, known liabilities
CHEMBL5082250	1 (MW > 500)	Yes	Non-mutagenic	Moderate risk	Strong candidate, needs validation
CHEMBL1651126	0	Yes	Non-mutagenic	Low risk	Promising lead
CHEMBL1651127	0	Yes	Non-mutagenic	Low risk	Promising lead
CHEMBL1651243	0	No	Mutagenic	High risk	Limited potential
CHEMBL310918	0	No	Mutagenic	High risk	Limited potential

Overall, the majority of compounds complied with Lipinski’s Rule of Five, suggesting favorable oral drug-likeness. A subset of molecules, including CHEMBL448008 (BACE1 reference inhibitor), CHEMBL1097353, CHEMBL1916158–1,916,170, CHEMBL5082250, and CHEMBL573106, showed violations primarily due to high molecular weight or excess hydrogen bond acceptors. Despite these deviations, several candidates maintained acceptable lipophilicity (iLogP values within the permissible range) and polar surface area parameters, supportive of oral bioavailability.

Of particular note, CHEMBL5082250 displayed only a single Lipinski violation (molecular weight > 500 Da) but retained balanced lipophilicity and favorable polar surface area values. These features are highly relevant for CNS drug discovery, as the ability to cross the BBB is indispensable for agents targeting AChE and BACE1. Indeed, BBB permeability predictions indicated that CHEMBL5082250, along with CHEMBL1651126, CHEMBL1651127, CHEMBL1651246, CHEMBL74359, CHEMBL74747, and CHEMBL76470, possesses physicochemical properties compatible with CNS penetration.

Toxicity predictions revealed marked heterogeneity across the ligand set. Most compounds were classified as non-mutagenic in the AMES test, whereas CHEMBL1651243 and CHEMBL310918 consistently exhibited toxicity liabilities across multiple endpoints, limiting their translational relevance. In contrast, CHEMBL1651126 and CHEMBL1651127 showed balanced ADMET properties with minimal violations, while CHEMBL5082250 was associated with moderate hepatotoxicity alerts and hERG inhibition risks. Interestingly, similar safety concerns were also observed for donepezil (CHEMBL502), the FDA-approved reference drug, suggesting that these liabilities, although noteworthy, may not necessarily preclude further development but will require rigorous experimental validation.

When compared with prior reports, our candidates exhibit profiles that are at least comparable, and in some cases superior, to previously proposed dual AChE/BACE1 inhibitors. For example, Banoo et al. [5] described indole–piperidine amides with favorable BBB permeability but reported multiple Lipinski violations, whereas our top-ranked ligands such as CHEMBL1651126 and CHEMBL1651127 satisfied all major oral drug-likeness criteria. Similarly, in Guzior et al. [20], several multifunctional anti-Alzheimer’s scaffolds demonstrated promising docking affinities but lacked systematic ADMET validation, a gap directly addressed in the present work. Notably, the balanced ADMET profiles of CHEMBL1651126/1651127 and the strong dual-target potential of CHEMBL5082250 suggest a more favorable translational trajectory compared with earlier candidates that often suffered from poor CNS penetration or high toxicity liabilities.

The integration of ADMET profiling with cheminformatics and SHAP-based SAR interpretation enabled the identification of candidates combining favorable pharmacokinetics with BBB penetration potential. Among them, CHEMBL5082250 emerged as the most compelling molecule, combining strong docking affinities with predicted CNS activity and tolerable safety liabilities. Similarly, CHEMBL1651126 and CHEMBL1651127 demonstrated promising drug-like properties with minimal toxicity concerns.

Taken together, these findings not only highlight the translational promise of selected candidates as dual AChE/BACE1 inhibitors but also position them within the broader context of Alzheimer’s drug discovery, where previous dual-inhibitor efforts often fell short in balancing efficacy with pharmacokinetics. While computational insights provide a rational foundation for lead prioritization and translational advancement, experimental validation through enzymatic inhibition assays, in vitro BBB permeability studies, and hepatotoxicity assessments will be essential to confirm their clinical applicability.

### Molecular docking results

Acetylcholinesterase (AChE) contains a deep and narrow active-site gorge that houses the catalytic triad (Ser203, Glu334, His447) and the oxyanion hole (Gly121, Gly122, Ala204), which collectively stabilize transition states during substrate hydrolysis. The peripheral anionic site (PAS), comprising residues such as Trp86, Tyr337, and Tyr341, further contributes to ligand recognition and substrate guidance [7]. In contrast, β-secretase 1 (BACE1) is an aspartyl protease with a catalytic dyad (Asp32, Asp228) situated within a wide cleft, where the conformationally dynamic flap region—formed by Tyr71, Trp76, and Phe108—regulates substrate accessibility and inhibitor binding [1]. These structural features define critical hot spots for inhibitor engagement and rationalize the application of molecular docking to identify high-confidence dual AChE/BACE1 inhibitors.

To validate the docking protocol, control ligands were redocked into their native binding pockets. Donepezil (CHEMBL502), the FDA-approved AChE inhibitor, yielded a docking score of – 10.45 kcal/mol (K_i_ = 21.79 nM), forming a hydrogen bond with Phe295 and additional π–π and hydrophobic contacts with Trp286, Tyr341, and Phe297 (Fig. 14). Similarly, the BACE1 reference inhibitor CHEMBL448008 produced a docking score of – 8.32 kcal/mol (K_i_ = 791.03 nM), stabilized by hydrogen bonds with Gln73, Lys107, Phe108, and Gly230, alongside π–cation interactions with Arg235 and van der Waals contacts within the catalytic cleft (Fig. 15). Both controls reproduced literature-supported interactions, confirming the robustness of the docking strategy. In both cases, the redocked poses reproduced the crystallographic orientations with RMSD values below 2.0 Å, further confirming the robustness of the docking strategy. Superpositions of the crystallographic ligands and their redocked poses are provided in Supplementary Figs. S1 and S2 to visually illustrate this agreement.

Several ligands demonstrated markedly stronger binding affinities compared to the reference inhibitors. Against AChE, binding energies ranged from − 9.03 to − 15.00 kcal/mol, with CHEMBL5082250 (− 15.00 kcal/mol, K_i_ = 10.06 pM), CHEMBL1651127 (− 13.21 kcal/mol, K_i_ = 206.44 pM), and CHEMBL1651126 (− 13.22 kcal/mol, K_i_ = 237.71 pM) emerging as top-scoring candidates. In parallel, BACE1 docking yielded binding energies between –8.32 and –14.51 kcal/mol, with the same three ligands consistently outperforming CHEMBL448008 (− 8.32 kcal/mol). Notably, CHEMBL1651126 and CHEMBL1651127 displayed balanced dual inhibition, with nanomolar affinities for both targets (Table 8).

**Table 8:** Binding energies and conventional H-bonding patterns of reference and candidate inhibitors against AChE and BACE1

CHEMBL ID	AChE			BACE1		
	Binding affinity (kcal/mol)	*K*_i_	Conventional H-Bonds	Binding affinity (kcal/mol)	*K*_i_	Conventional H-bonds
^a^CHEMBL502	− 10.45	21.79 nM	PHE295	− 10.37	25.02 nM	THR72, GLN73
^b^CHEMBL448008	− 9.50	108.37 nM	TYR124, HIS447	− 8.32	791.03 nM	GLN73, LYS107, PHE108, GLY230, THR232
CHEMBL1083662	− 13.37	159.21 pM	PHE295	− 11.19	6.22 nM	THR72
CHEMBL1097353	− 12.67	517.76 pM	TYR124, ARG296	− 12.19	1.17 nM	THR72, GLN73, GLY230
CHEMBL1651126	− 13.22	237.71 pM	SER293, PHE295	− 11.03	8.29 nM	THR72, GLN73
CHEMBL1651127	− 13.21	206.44 pM	TYR72, PHE295	− 11.08	7.50 nM	THR72, PHE108
CHEMBL1651243	− 12.64	542.27 pM	GLY121, GLY122, PHE295, HIS447	− 12.23	1.08 nM	THR72, ARG128, THR232, ASN233
CHEMBL1651246	− 12.95	322.67 pM	SER293, TYR341	− 11.39	4.47 nM	THR72, ASP228
CHEMBL1916158	− 9.74	72.03 nM	TYR124, TRP286, SER293, PHE295, ARG296	− 10.01	46.15 nM	GLY11, GLN73, ASP228, THR231, ARG307
CHEMBL1916166	− 13.40	151.56 pM	GLY121, GLY122, ARG296	− 10.50	20.09 nM	ASP32, GLN73, ARG128, TYR198, THR232
CHEMBL1916167	− 9.72	74.61 nM	GLY121, GLY122, ARG296	− 14.51	22.95 pM	GLN73, ASP228, THR231, THR232, ASN233
CHEMBL1916168	− 10.04	43.55 nM	SER293, ARG296, TYR341	− 11.61	3.07 nM	ASP32, GLN73, ARG128, GLY230
CHEMBL1916169	− 10.84	11.27 nM	TYR72, THR75	− 11.42	4.24 nM	GLY11, GLN73, ASP228, GLY230, THR232
CHEMBL1916170	− 12.28	1.00 nM	TYR72, THR75, TYR124	− 10.52	19.57 nM	ASP32, THR72, GLY230, THR232
CHEMBL310918	− 11.97	1.69 nM	GLY121, GLY122, SER293, PHE295	− 10.68	14.73 nM	SER36, ILE126, TYR198, THR232
CHEMBL3922159	− 13.85	70.91 pM	ASP74	− 9.77	68.86 nM	GLY11, GLN73, THR232
CHEMBL3973939	− 12.21	1.11 nM	ASP74, GLY126	− 9.03	241.31 nM	GLY230, THR232
CHEMBL5082250	− 15.00	10.06 pM	GLY120, ARG296, TYR341	− 13.27	187.09 pM	SER36, ASN37, GLN73
CHEMBL573106	− 10.21	32.66 nM	TYR72, THR75, TYR124, PHE295	− 8.75	384.04 nM	GLY11, GLY230, THR232, ASN233
CHEMBL74359	− 11.61	3.11 nM	PHE295, TYR341	− 10.32	27.09 nM	THR72
CHEMBL74747	− 12.16	1.22 nM	PHE295	− 10.67	15.10 nM	THR72, GLN73
CHEMBL76470	− 11.71	2.60 nM	PHE295	− 10.02	45.58 nM	THR72, GLN73

^a^Donepezil—AChE reference inhibitor and FDA-approved drug

^b^BACE1 reference inhibitor

**Fig. 13:**
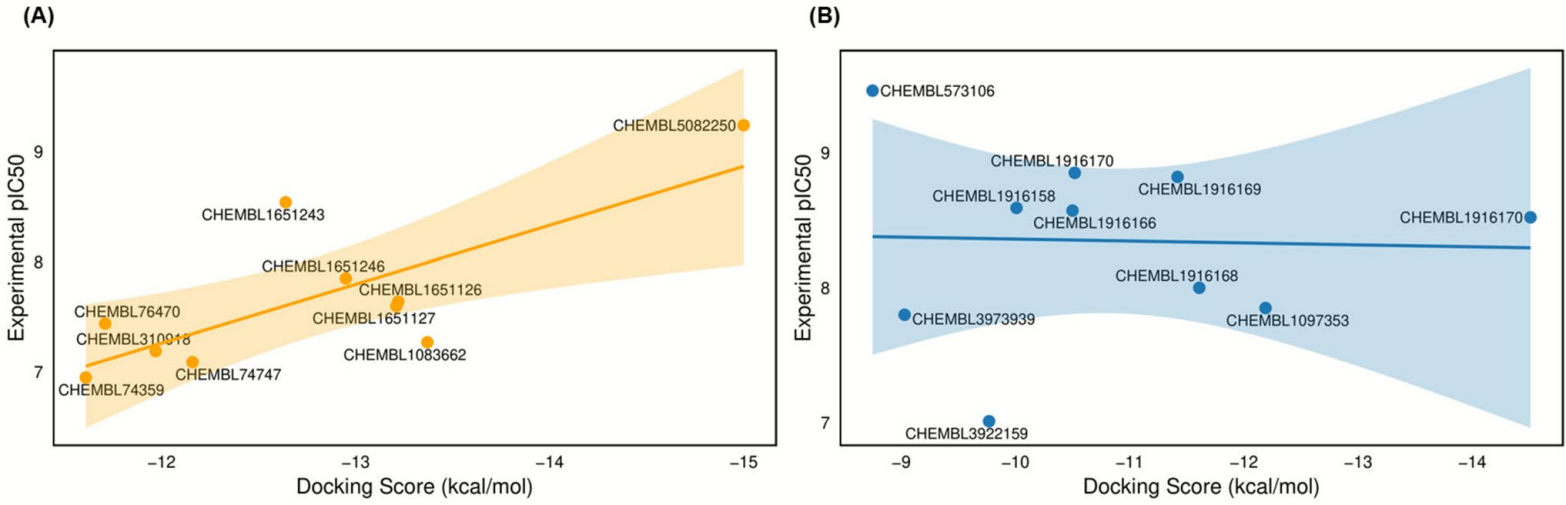
Correlation plots between docking scores and experimental activity values (pIC_50_) for AChE (**A**) and BACE1 (**B**)

Correlation analysis between docking scores and experimental activity values (experimental pIC_50_ values listed in Table 6) revealed a moderately strong and statistically significant inverse association for AChE (r = − 0.76, R^2^ = 0.58, *p* = 0.010; n = 10), indicating a reasonable level of consistency between calculated binding affinities and inhibitory potency for this target. This suggests that rigid-receptor docking can qualitatively reflect the relative activity trends of AChE ligands. In contrast, no meaningful correlation was observed for BACE1 (r = 0.04, R^2^ = 0.001, *p* = 0.923; n = 10), which aligns with the known structural flexibility and large catalytic pocket of the enzyme that limit the quantitative predictive capacity of conventional docking approaches (Fig. 13).

**Fig. 14:**
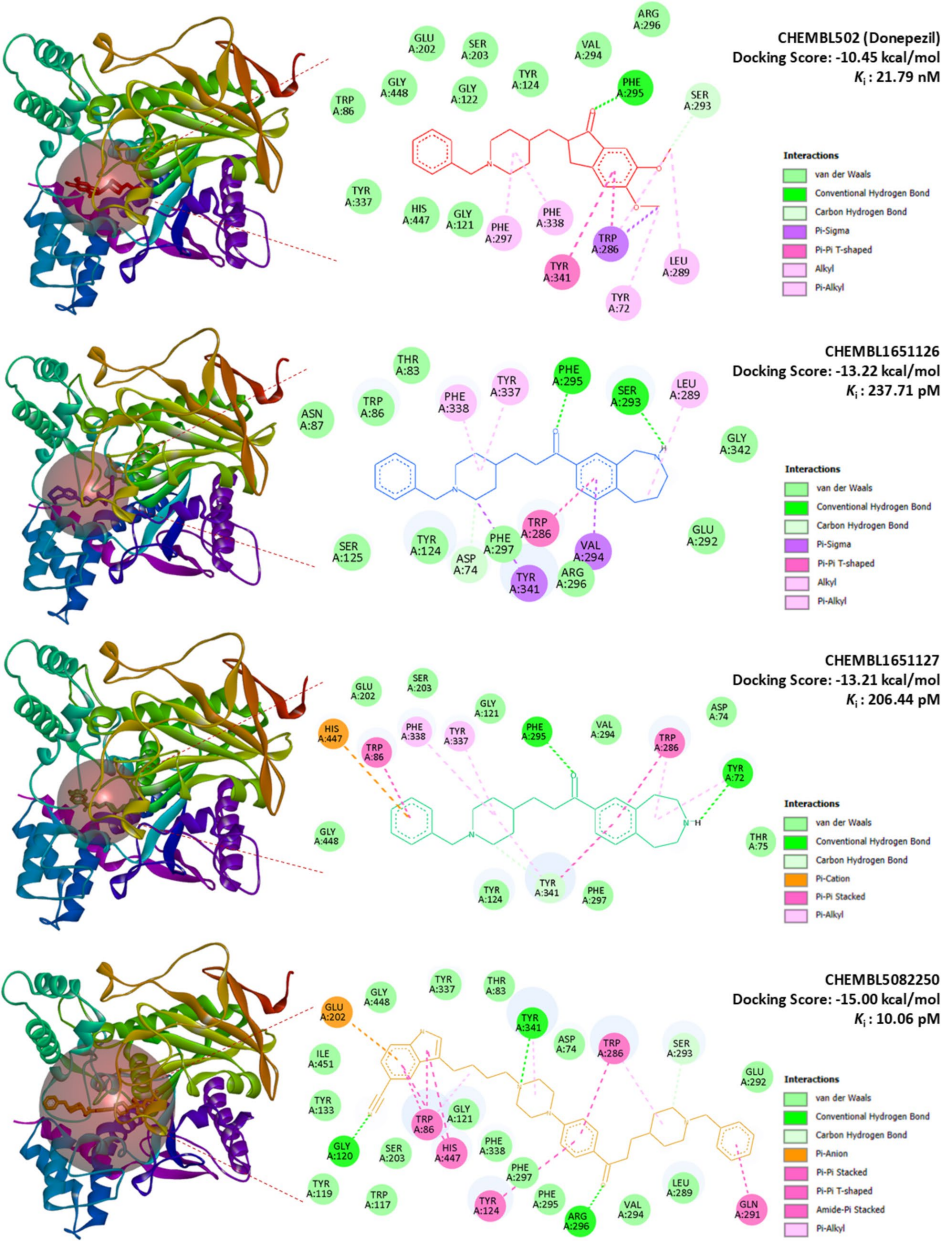
Docking poses and interaction maps of AChE inhibitors. Docking poses (left) and 2D interaction maps (right) for the reference ligand CHEMBL502 (Donepezil) and the top-scoring candidates CHEMBL1651126, CHEMBL1651127, and CHEMBL5082250 within the acetylcholinesterase (AChE) binding site. Docking scores and predicted Ki values are shown for each compound. Interaction types are color coded: conventional hydrogen bonds (green), carbon hydrogen bonds (light green), π–π stacked/T-shaped and amide–π interactions (magenta), π–alkyl interactions (violet), π–anion interactions (orange), and van der Waals contacts (pale green)

**Fig. 15:**
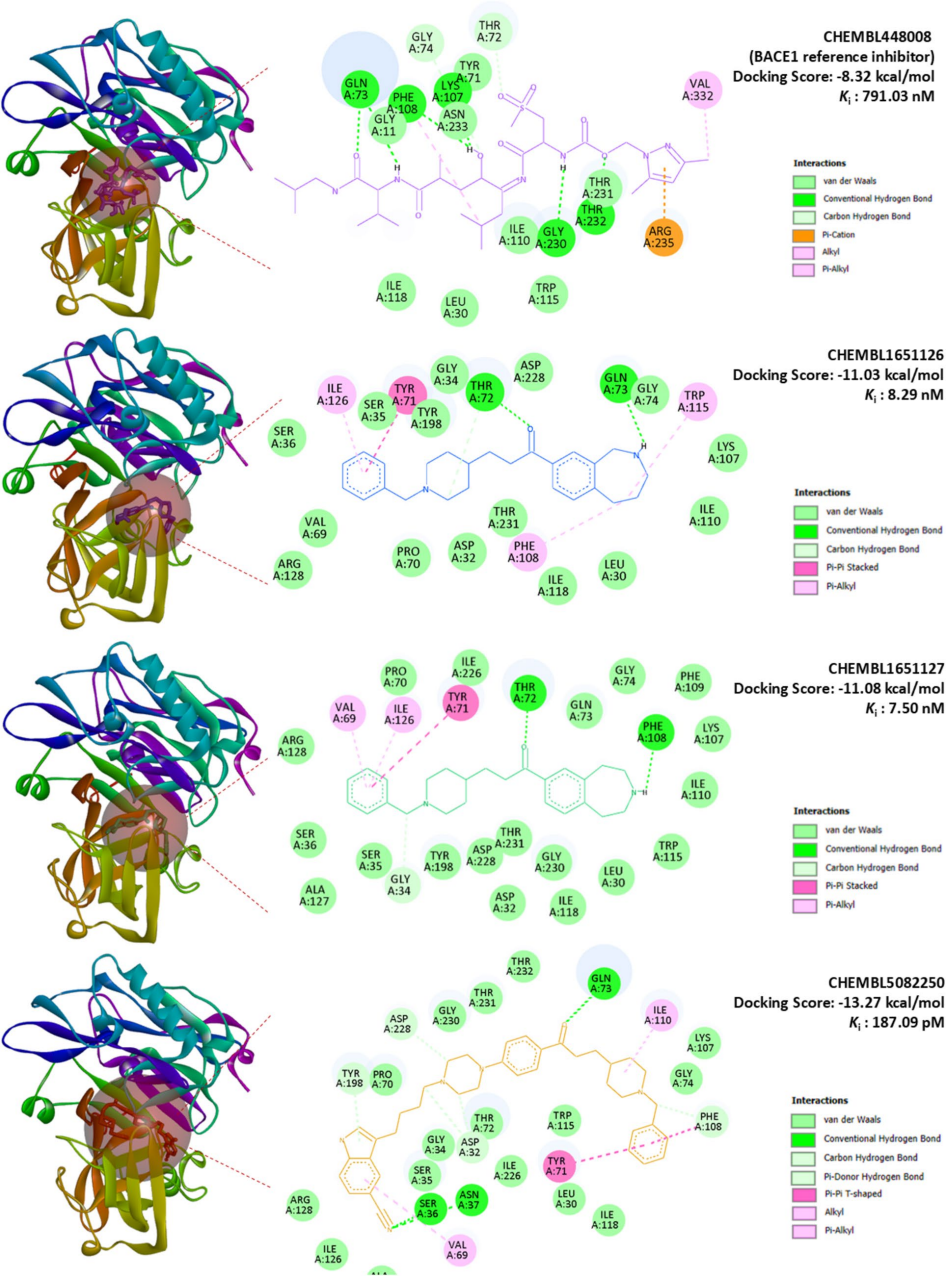
Docking poses and interaction maps of BACE1 inhibitors. Docking poses (left) and 2D interaction maps (right) for the reference inhibitor CHEMBL448008 and the top-scoring candidates CHEMBL1651126, CHEMBL1651127, and CHEMBL5082250 within the β-secretase 1 (BACE1) binding site. Docking scores and predicted Ki values are shown for each compound. Interaction types are color coded: conventional hydrogen bonds (green), carbon hydrogen bonds (light green), π–π stacked/T-shaped interactions (magenta), π–alkyl interactions (violet), alkyl interactions (gray), and van der Waals contacts (pale green)

Interaction analysis revealed that CHEMBL5082250 exhibited the most extensive interaction network. Within AChE, it formed multiple hydrogen bonds with Gly120, Arg296, and Tyr341, π–π stacking with Trp86/Trp286, and hydrophobic contacts with Phe295/Phe338, collectively anchoring the ligand deep inside the catalytic gorge (Fig. 14). In BACE1, it engaged Ser36, Asn37, and Gln73 via hydrogen bonding, reinforced by π–π stacking with Tyr71 and carbon hydrogen bonding to Phe108, consistent with a highly stable dual-binding pose (Fig. 15).

CHEMBL1651126 and CHEMBL1651127 showed similarly favorable profiles: both formed hydrogen bonds with catalytically relevant residues in AChE (Ser293, Phe295, Tyr72) and BACE1 (Thr72, Gln73, Phe108), alongside stabilizing hydrophobic contacts. These recurrent binding patterns highlight a conserved mechanism of action across both enzymes.

When compared with previous docking studies, our ligands demonstrated notably stronger affinities. For example, Atta et al. [1] reported BACE1 inhibitors with binding energies in the − 7 to − 10 kcal/mol range, whereas CHEMBL5082250 reached − 13.27 kcal/mol against BACE1. Similarly, Bagrowska et al. [7] highlighted Donepezil’s stabilizing interactions within AChE, which our candidates not only reproduced but exceeded in terms of interaction diversity and binding strength. These findings indicate that the prioritized scaffolds in this work surpass the binding performance of several reported dual inhibitors, underscoring their potential as next-generation chemotypes.

From a drug discovery perspective, the identification of high-affinity multitarget ligands is particularly significant for Alzheimer’s disease, where simultaneous inhibition of AChE (symptomatic relief) and BACE1 (disease modification) may offer synergistic therapeutic benefits. The superior docking affinities of CHEMBL5082250, CHEMBL1651126, and CHEMBL1651127, combined with their predicted blood–brain barrier permeability and overall favorable ADMET profiles, underscore their translational potential as CNS-active agents. Importantly, while hepatotoxicity and hERG inhibition risks were flagged in silico for some candidates, comparable liabilities were also predicted for Donepezil, suggesting that such alerts warrant careful experimental validation rather than outright exclusion.

Taken together, our docking results not only validate the computational strategy but also extend current knowledge by identifying ligands with stronger and more diverse binding networks than those previously reported in the literature. Collectively, the docking outcomes highlight CHEMBL5082250 as the most potent dual inhibitor, supported by extensive interaction networks and subnanomolar binding predictions, with CHEMBL1651126 and CHEMBL1651127 emerging as balanced and promising alternatives. These findings not only provide structural insights into ligand–target recognition but also establish a rational framework for prioritizing candidates in subsequent in vitro enzymatic assays, BBB permeability models, and toxicity evaluations, thereby bridging computational predictions with clinical applicability. Thus, our results complement and extend the findings of Dhamodharan & Mohan by providing a more holistic pipeline for multi-target drug design, including structural binding, pharmacokinetics, and model interpretability.

## Conclusion

In this study, we established an integrative and transparent computational framework that couples explainable QSAR modeling with molecular docking and ADMET profiling to prioritize multitarget inhibitors of AChE and BACE1—two central enzymes implicated in Alzheimer’s disease pathology. Rigorous dataset curation, scaffold-based validation, and applicability domain analyses ensured model robustness and minimized bias, while SHAP-driven interpretation provided chemically intuitive insights into the structural determinants of dual inhibition.

Our results highlight several promising scaffolds, notably CHEMBL5082250, CHEMBL1651126, and CHEMBL1651127, which consistently demonstrated high predictive probabilities, favorable docking poses, and physicochemical properties compatible with CNS penetration. These compounds not only reproduced key interactions within the catalytic regions of AChE and BACE1 but also achieved superior binding affinities compared to reference inhibitors. Importantly, their pharmacokinetic and toxicity profiles suggest translational potential, although moderate hepatotoxicity and hERG liability signals underscore the necessity of experimental validation.

By integrating predictive performance with mechanistic interpretability, this work advances beyond conventional black-box screening approaches, delivering a reproducible pipeline that can accelerate the rational discovery of dual-acting chemotypes. Future directions should involve enzymatic inhibition assays, in vitro BBB models, and in vivo pharmacodynamic studies to validate the computationally prioritized scaffolds. Collectively, our findings underscore the value of combining explainable machine learning with molecular modeling in the pursuit of next-generation, disease-modifying therapeutics for Alzheimer’s disease.

## Supplementary Information

Below is the link to the electronic supplementary material.

Supplementary file1 (DOCX 582 KB)

## Data Availability

No datasets were generated or analysed during the current study.Code availability The code scripts and trained models used in this study are available from the corresponding author upon reasonable request.

## Code availability

The code scripts and trained models used in this study are available from the corresponding author upon reasonable request.
