# Supplementary material for: Computational prioritization of multi-target inhibitors: explainable QSAR and docking-based discovery of dual AChE/BACE1 chemotypes
Source: J Comput Aided Mol Des. 2026 Jan 28;40(1):54. doi: 10.1007/s10822-025-00757-3 (PMC12852253; doi:10.1007/s10822-025-00757-3)
Supplement: Supplementary file 1 — Supplementary file1 (DOCX 582 KB) [file 10822_2025_757_MOESM1_ESM.docx]

**Supplementary Table S1.** Top 20 ECFP6 bits contributing to the **GBDT–ECFP6** classification model for dual AChE–BACE1 inhibition, ranked by mean absolute SHAP value. Columns: Bit ID, Mean |SHAP|, Mean SHAP (bit=1), Mean SHAP (bit=0), Direction.

| **Bit ID** | **Mean \|SHAP\|** | **Mean SHAP (bit=1)** | **Mean SHAP (bit=0)** | **Direction** |
| --- | --- | --- | --- | --- |
| ECFP_680 | 0.817 | 1.528 | -0.588 | **Positive SHAP effect** |
| ECFP_738 | 0.748 | 0.99 | -0.593 | **Positive SHAP effect** |
| ECFP_1162 | 0.735 | -2.509 | 0.23 | Negative SHAP effect |
| ECFP_1928 | 0.728 | 0.937 | -0.652 | **Positive SHAP effect** |
| ECFP_1321 | 0.714 | 4.673 | -0.285 | **Positive SHAP effect** |
| ECFP_1367 | 0.659 | 2.284 | -0.531 | **Positive SHAP effect** |
| ECFP_1599 | 0.534 | -3.631 | 0.199 | Negative SHAP effect |
| ECFP_896 | 0.532 | 1.06 | -0.355 | **Positive SHAP effect** |
| ECFP_864 | 0.506 | -1.565 | 0.288 | Negative SHAP effect |
| ECFP_661 | 0.494 | 3.914 | -0.409 | **Positive SHAP effect** |
| ECFP_1412 | 0.4 | -3.721 | 0.138 | Negative SHAP effect |
| ECFP_1602 | 0.399 | -0.848 | 0.191 | Negative SHAP effect |
| ECFP_1181 | 0.396 | -2.423 | 0.176 | Negative SHAP effect |
| ECFP_1143 | 0.38 | -2.048 | 0.2 | Negative SHAP effect |
| ECFP_807 | 0.362 | -0.32 | 0.381 | Negative SHAP effect |
| ECFP_29 | 0.273 | 2.484 | -0.16 | **Positive SHAP effect** |
| ECFP_1504 | 0.209 | 1.676 | -0.093 | **Positive SHAP effect** |
| ECFP_809 | 0.19 | 1.441 | -0.091 | **Positive SHAP effect** |
| ECFP_701 | 0.156 | -2.876 | 0.088 | Negative SHAP effect |
| ECFP_1920 | 0.154 | -1.286 | 0.065 | Negative SHAP effect |

**Supplementary Table S2**. In silico ADMET predictions of the ligand dataset obtained from SwissADME and pkCSM.

|  | **CHEMBL76470** | 376.53 g/mol | 6 | 2 | 0 | 3,97 | 23.55 Å² | 124.27 | -2.06 | Yes; 0 violation | Safe (High Confidence) | Safe (Low Confidence) | Toxic (Medium Confidence) | Toxic (High Confidence) |
| --- | --- | --- | --- | --- | --- | --- | --- | --- | --- | --- | --- | --- | --- | --- |
|  | **CHEMBL74747** | 390.56 g/mol | 6 | 2 | 0 | 4,28 | 23.55 Å² | 129.08 | -2.16 | Yes; 0 violation | Safe (High Confidence) | Safe (Low Confidence) | Toxic (Low Confidence) | Toxic (High Confidence) |
|  | **CHEMBL74359** | 348.48 g/mol | 6 | 2 | 1 | 3,58 | 32.34 Å² | 114.56 | -2.00 | Yes; 0 violation | Safe (High Confidence) | Safe (Low Confidence) | Toxic (Medium Confidence) | Toxic (High Confidence) |
|  | **CHEMBL573106** | 851.95 g/mol | 24 | 11 | 5 | 4,97 | 200.85 Å² | 221.25 | -2.98 | No; 2 violations: MW>500, NorO>10 | Safe (High Confidence) | Safe (Low Confidence) | Toxic (High Confidence) | Toxic (High Confidence) |
|  | **CHEMBL5082250** | 587.80 g/mol | 12 | 4 | 1 | 5,24 | 66.37 Å² | 191.21 | -2.05 | Yes; 1 violation: MW>500 | Safe (High Confidence) | Safe (Medium Confidence) | Toxic (Medium Confidence) | Toxic (High Confidence) |
|  | **CHEMBL3973939** | 475.60 g/mol | 6 | 4 | 1 | 3,99 | 67.92 Å² | 142.06 | -3,02 | Yes; 1 violation: MLOGP>4.15 | Safe (High Confidence) | Safe (Low Confidence) | Toxic (Low Confidence) | Toxic (High Confidence) |
|  | **CHEMBL3922159** | 471.63 g/mol | 6 | 3 | 1 | 4,21 | 67.92 Å² | 146.91 | -3,07 | Yes; 1 violation: MLOGP>4.15 | Safe (High Confidence) | Safe (Low Confidence) | Safe (Low Confidence) | Toxic (High Confidence) |
|  | **CHEMBL310918** | 393.48 g/mol | 7 | 4 | 1 | 3,17 | 78.16 Å² | 123.38 | -2.38 | Yes; 0 violation | Toxic (High Confidence) | Toxic (High Confidence) | Toxic (High Confidence) | Toxic (High Confidence) |
|  | **CHEMBL1916170** | 705.83 g/mol | 18 | 9 | 4 | 4,72 | 132.89 Å² | 196.83 | -2.74 | Yes; 1 violation: MW>500 | Safe (High Confidence) | Safe (Medium Confidence) | Toxic (High Confidence) | Toxic (High Confidence) |
|  | **CHEMBL1916169** | 705.83 g/mol | 18 | 9 | 4 | 4,35 | 132.89 Å² | 196.83 | -2.60 | Yes; 1 violation: MW>500 | Safe (High Confidence) | Safe (Medium Confidence) | Toxic (High Confidence) | Toxic (High Confidence) |
|  | **CHEMBL1916168** | 722.84 g/mol | 18 | 9 | 4 | 5,23 | 120.00 Å² | 198.99 | -2,71 | Yes; 1 violation: MW>500 | Safe (High Confidence) | Safe (Medium Confidence) | Toxic (Medium Confidence) | Toxic (High Confidence) |
|  | **CHEMBL1916167** | 729.86 g/mol | 18 | 9 | 4 | 5,22 | 143.79 Å² | 203.75 | -2.37 | Yes; 1 violation: MW>500 | Safe (High Confidence) | Safe (Medium Confidence) | Toxic (High Confidence) | Toxic (High Confidence) |
|  | **CHEMBL1916166** | 772.84 g/mol | 19 | 11 | 4 | 5,3 | 120.00 Å² | 204.03 | -2.83 | Yes; 1 violation: MW>500 | Safe (High Confidence) | Safe (Medium Confidence) | Toxic (High Confidence) | Toxic (High Confidence) |
|  | **CHEMBL1916158** | 691.81 g/mol | 17 | 9 | 4 | 5,16 | 132.89 Å² | 192.46 | -2.91 | Yes; 1 violation: MW>500 | Safe  (High Confidence) | Safe  (Medium Confidence) | Toxic (High Confidence) | Toxic (High Confidence) |
|  | **CHEMBL1651246** | 413.53 g/mol | 7 | 5 | 1 | 2,96 | 83.82 Å² | 120.08 | -2,55 | Yes; 0 violation | Safe  (High Confidence) | Safe (Low Confidence) | Toxic (Medium Confidence) | Toxic (High Confidence) |
|  | **CHEMBL1651243** | 445.51 g/mol | 7 | 5 | 0 | 3,46 | 88.13 Å² | 133.13 | -2.47 | Yes; 0 violation | Toxic (High Confidence) | Toxic (High Confidence) | Toxic (High Confidence) | Toxic (High Confidence) |
|  | **CHEMBL1651127** | 376.53 g/mol | 6 | 3 | 1 | 3,82 | 32.34 Å² | 123.62 | -2.46 | Yes; 0 violation | Safe  (High Confidence) | Safe (Medium Confidence) | Toxic (Low Confidence) | Toxic (High Confidence) |
|  | **CHEMBL1651126** | 376.53 g/mol | 6 | 3 | 1 | 3,92 | 32.34 Å² | 123.62 | -2.55 | Yes; 0 violation | Safe  (High Confidence) | Safe (Medium Confidence) | Toxic (Low Confidence) | Toxic (High Confidence) |
|  | **CHEMBL1097353** | 684.79 g/mol | 14 | 10 | 3 | 4,8 | 136.66 Å² | 188.49 | -3.37 | Yes; 1 violation: MW>500 | Safe  (High Confidence) | Safe  (High Confidence) | Toxic (High Confidence) | Toxic (High Confidence) |
|  | **CHEMBL1083662** | 438.60 g/mol | 7 | 2 | 0 | 4,48 | 23.55 Å² | 144.51 | -1.73 | Yes; 1 violation: MLOGP>4.15 | Safe  (High Confidence) | Safe (Low Confidence) | Toxic (Medium Confidence) | Toxic (High Confidence) |
|  | **CHEMBL448008** (BACE1 reference inhibitor) | 658.85 g/mol | 23 | 9 | 5 | 3,91 | 206.20 Å² | 172.40 | -2.50 | No; 2 violations: MW>500, NorO>10 | Safe  (High Confidence) | Safe  (High Confidence) | Toxic (High Confidence) | Safe  (High Confidence) |
|  | **CHEMBL502** (Donepezil – AChE reference inhibitor and FDA-approved drug) | 379.49 g/mol | 6 | 4 | 0 | 3,92 | 38.77 Å² | 115.31 | -2.33 | Yes; 0 violation | Safe  (High Confidence) | Safe (Medium Confidence) | Toxic (Low Confidence) | Toxic (High Confidence) |
|  |  | **Molecular weight (g/mol)** | **Rotable bond** | **H-bond acceptor** | **H-bond donor** | **iLogP** | **Polar Surface Area** | **Molar Refractivity** | **BBB permeability** | **Lipinski Rule** | **AMES Mutagenesis** | **Liver Injury I** | **Liver Injury II** | **hERG Blockers** |


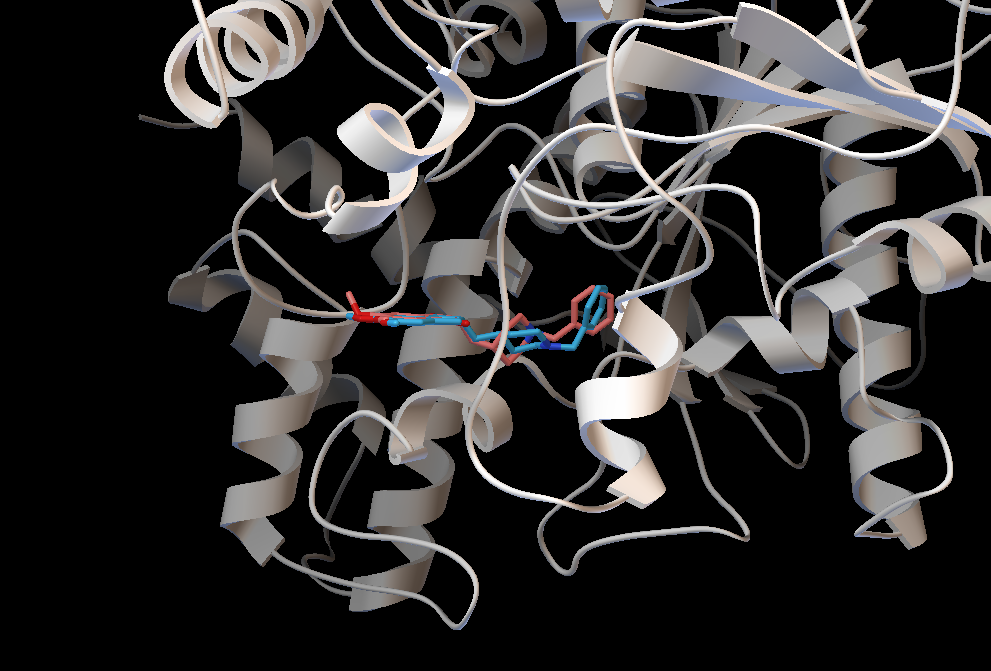


**Supplementary Figure S1.** Superposition of the crystallographic pose and the redocked pose of donepezil (CHEMBL502) in the AChE active site. The protein structure is shown in grey. The blue ligand represents the original crystallographic orientation retrieved from the PDB structure, while the orange ligand depicts the redocked pose generated in this study.


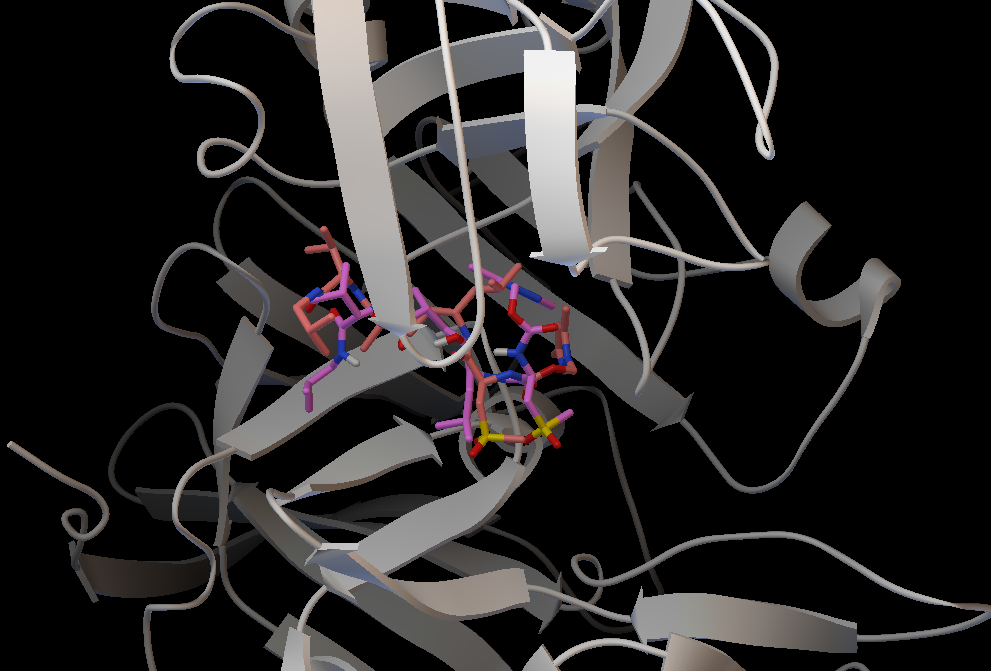


**Supplementary Figure S2.** Superposition of the crystallographic pose and the redocked pose of reference inhibitor (CHEMBL448008) in the BACE1 active site. The protein structure is shown in grey. The orange ligand represents the original crystallographic orientation retrieved from the PDB structure, while the purple ligand depicts the redocked pose generated in this study.
